# Genomic insights into longan evolution from a chromosome-level genome assembly and population genomics of longan accessions

**DOI:** 10.1093/hr/uhac021

**Published:** 2022-02-19

**Authors:** Jing Wang, Jianguang Li, Zaiyuan Li, Bo Liu, Lili Zhang, Dongliang Guo, Shilian Huang, Wanqiang Qian, Li Guo

**Affiliations:** 1 Key Laboratory of South Subtropical Fruit Biology and Genetic Resource Utilization, Ministry of Agriculture, Key Laboratory of Tropical and Subtropical Fruit Tree Research of Guangdong Province, Guangzhou, China; 2 Institution of Fruit Tree Research, Guangdong Academy of Agricultural Sciences, Guangzhou, China; 3 Agricultural Genomics Institute at Shenzhen, Chinese Academy of Agricultural Sciences, Shenzhen, China; 4 Weifang Institute of Technology, Weifang, China; 5 Peking University Institute of Advanced Agricultural Sciences, Weifang, China; 6 Faculty of Electronic and Information Engineering, Xi’an Jiaotong University, Xi’an, China

## Abstract

Longan (*Dimocarpus longan*) is a subtropical fruit tree best known for its nutritious fruit and regarded as a valuable tonic and traditional medicine since ancient times. A high-quality chromosome-scale genome assembly is valuable for functional genomic study and genetic improvement of longan. Here, we report a chromosome-level reference genome sequence for the longan cultivar JDB. The assembled genome is 455.5 Mb in size and anchored to fifteen chromosomes, representing a significant improvement in contiguity (contig N50 = 12.1 Mb, scaffold N50 = 29.5 Mb) over a previous draft assembly. A total of 40 420 protein-coding genes were predicted in the *D. longan* genome. Synteny analysis suggests that longan shares the widespread gamma event with core eudicots but has no other whole genome duplications. Comparative genomics showed that the *D. longan* genome experienced significant expansions of UDP-glucosyltransferase and phenylpropanoid biosynthesis-related gene families. Deep genome sequencing analysis of longan cultivars identified longan biogeography as a major contributing factor to its genetic diversity and revealed clear population admixture and introgression among cultivars of different geographic origins, suggesting a likely migration trajectory of longan that is confirmed by existing historical records. Finally, genome-wide association studies (GWAS) of longan cultivars identified quantitative trait loci (QTLs) for six different fruit quality traits and revealed a shared QTL that contained three genes for total soluble solids and seed weight. The chromosome-level reference genome assembly, annotation, and population genetic resources for *D. longan* will facilitate the molecular studies and breeding of desirable longan cultivars in the future.

## Introduction

Longan (*D. longan* Lour.), also known as dragon’s eyeball and closely related to lychee, is a tropical/subtropical evergreen fruit tree in the Sapindaceae family with a diploid genome [1] (2n = 2x = 30). It is regarded as a valuable tonic and has traditionally been used as a medicinal plant with rich pharmaceutical effects from many plant parts, especially fruits, and it contributes to rural economic development in tropical and subtropical areas. The use of longan in traditional herbal remedies was recorded in the compendium of materia medica (Ben Cao Gang Mu in Chinese) by Li Shizhen, a famous traditional Chinese medicine expert during the Ming Dynasty, who called longan the king of fruits [2]. Given its high nutritional and economic value, longan is cultivated in many countries such as China, Australia, Thailand, and Vietnam. Among these countries, China has the highest production of longan with the largest cultivation area [3], including Guangdong, Guangxi, Fujian, Hainan, and other regions [4].

China has a long history of longan cultivation and use. Native to South China, longan has been cultivated in China for over 2000 years with rich germplasm resources [5]. Abundant wild resources are found in Yunnan and Hainan provinces, whence longan was introduced to South Asian countries such as Thailand [6]. A previous study based on the different pollen exine patterns of fourteen longan varieties supports Yunnan as the primary center of longan origin and Guangdong, Guangxi, and Hainan as the secondary centers [7]. To date, the population genetic structure of longan remains poorly characterized. Longan varieties have a rather ambiguous genetic background owing to the fact that they reproduce by both inbreeding and crossbreeding in the field. A previous analysis using ISSR (inter-simple sequence repeat) markers indicated that Thai and Vietnamese varieties have close genetic relationships [8]. However, the classification of longan varieties using these markers has been inconclusive because of differences in marker selection, number of varieties, and classification methods. A resolved population structure of longan varieties and a better understanding of its genetic diversity and migration history are essential for its conservation and breeding but are not yet available, as they require a large-scale phylogenomic study of longan varieties from around China and Southeast Asia.

Longan leaves, flowers, fruits, and seeds [9, 10] are rich in polyphenols with anti-cancer and anti-oxidant properties that are biosynthesized primarily through the shikimic acid, flavonoid, and phenylpropanoid pathways. The phenylpropanoid pathway is one of the most extensively investigated specialized metabolic pathways. Branches of the phenylpropanoid pathway produce metabolites such as flavonoids, hydroxycinnamic acid esters, hydroxycinnamic acid amides, and the precursors of lignin, lignans, and tannins. These phenolic natural products are key components of the nutritive, flavor, and medicinal properties of longan. Phenylpropanoids also play important roles in plant resistance to pathogen infection [11] either by acting as physical barriers against invasion or through chemical toxicity to herbivores and microbial pathogens. Therefore, these secondary metabolites are acquired traits that offer adaptive advantages to longan through evolution and can be exploited by humans as useful medicines. Despite the vital roles of phenylpropanoids in the nutrition and flavor of longan fruits, their biosynthetic pathways in longan remain uncharacterized because of limited genetic and genomic resources and the technical difficulty of genetic transformation, which impede the improvement of desired traits in longan fruits through molecular and genomic breeding.

Longan breeding, which relies mainly on sexual hybridization, typically targets two main traits: fruit size and sweetness [12]. Longan breeding is challenging and time-consuming because of its long juvenile period and the difficulty of genetic transformation. Marker-assisted selection (MAS) is an effective biotechnological tool that enables early selection of hybrid progenies at the seedling stage [13]. However, our knowledge about the genetic mapping of longan is limited. Guo *et al.* (2011) constructed a low-quality male and female genetic map consisting of 243 and 184 separate molecular markers [14]. Single nucleotide polymorphism (SNP) markers based on restriction site-associated DNA sequencing (RAD-seq) were developed for QTL identification using hybrid F_1_ progenies and their two parents as materials [13]. A high-quality reference genome sequence and information on the longan genetic background would significantly facilitate the investigation of genotype–phenotype associations of longan germplasms and thus expedite the longan breeding program. Although a draft genome sequence of the *D. longan* “HHZ” cultivar is available [9], the assembly is highly fragmented, composed of 51 392 contigs with a contig N50 of 26 kb.

Here, we produced a chromosome-level genome assembly for the *D. longan* JDB cultivar by combining Illumina paired-end (PE), PacBio single molecule real-time (SMRT), and high-throughput chromatin conformation capture (Hi-C) sequencing. We annotated the genome using *ab initio* prediction, homolog evidence, and multi-tissue transcriptomic data. In addition, we performed population genome sequencing from a collection of longan accessions, followed by an in-depth analysis of population structure using high-quality genetic variants. The analysis revealed the population genetic diversity of longan and demonstrated population admixture and introgression among cultivars from major longan growing areas. GWAS analysis revealed three genes that were associated with fruit total soluble solids and seed weight, suggesting parallel evolution in the evolutionary diversification of domesticated species. The genome assembly, annotations, and genetic variants are valuable for functional genomic studies as well as molecular breeding of *D. longan* to improve yield and fruit quality and exploit its medicinal properties.

## Results

### Genome assembly and annotation

**Figure 1:**
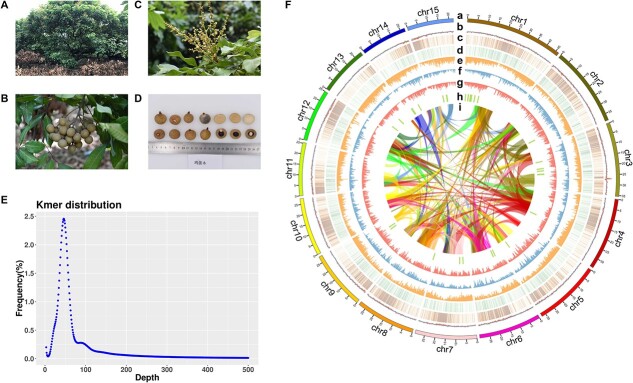
**Chromosome-level genome assembly of longan (*Dimocarpus longan* Lour.).** (**A–D**): Photos of tree (**A**), flower (**B**), fruit cluster (**C**), and fruit section (**D**) of the longan cultivar JDB. (**E**) Kmer frequency distribution analysis for the JDB genome based on Illumina paired-end reads. (**F**) Overview of the *D. longan* genome. Tracks a to i: chromosomes, GC-content, density of *Gypsy* LTRs, density of *Copia* LTRs, density of protein-coding genes, SNP density, Indel density, distribution of secondary metabolic gene clusters (predicted using *plantiSMASH*), and syntenic blocks (colored ribbons). The density statistics were calculated within genomic windows 150 kb in size.

The *D. longan* cultivar “JDB” originated in Fujian and is planted in the Longan Germplasm Repository of Guangdong Province (Figure 1A-1D); fresh young leaves were collected for genomic DNA isolation and sequencing. To construct a chromosome-level reference genome for *D. longan*, a total of 184.4 Gb PacBio SMRT reads (~415× coverage), 25.3 Gb (~56× coverage) Illumina PE reads, and 57.6 Gb (~127× coverage) Hi-C Illumina read pairs were generated (Supplementary Table 1). We estimated the genome size of *D. longan* cultivar JDB as 474.98 Mb with a heterozygosity rate of 0.36% via the distribution of k-mer frequency using Illumina PE reads (Figure 1E). PacBio SMRT reads were used to assemble the *D. longan* genome with *Canu*^15^, followed by polishing contigs with *Pilon*^16^ using Illumina PE reads, yielding a draft genome assembly of 455.5 Mb (Table 1). Next, Hi-C paired-end reads were used to anchor the contigs to chromosomes with *3D-DNA* [17]. The final *D. longan* JDB genome assembly of 455.5 Mb covers 95.90% of the estimated genome size (474.98 Mb), and 98.7% of the assembled sequences are anchored onto 15 chromosomes (Figure 1F; Supplementary Figure 1) with contig and scaffold N50s of 12.1 Mb and 29.6 Mb, respectively (Table 1). Thus, this longan genome assembly represents a significant improvement over the highly fragmented *D. longan* HHZ genome assembly (contig N50: 0.026 Mb) released previously [9]. Genome completeness was assessed using the plant dataset of the Benchmarking Universal Single Copy Orthologs (BUSCO) database v1.22 [18] with e-value <1e−5. BUSCO evaluation revealed the 98.1% completeness of our *D. longan* genome assembly (88.4% single copy, 9.7% duplicated copy, 1.1% fragmented, and 0.8% missing) (Table 1, Supplementary Table 2).

**Table 1:** Statistics for *Dimocarpus longan* JDB genome assembly and annotations

	**Statistics**	***D. longan* JDB (this study)**	***D. longan* Honghezi** ^**9**^
**Contig**	Total number of contigs	250	51 392
	Assembly size (Mb)	455.5	471.9
	Contig N50 (Mb)	12.1	0.026
	Contig N90 (Mb)	1.8	0.006
	Largest Contig (Mb)	31.1	0.17
**Scaffold**	Total number of scaffolds	90	17 367
	Assembly size (Mb)	455.5	495.3
	Scaffold N50 (Mb)	29.6	0.57
	Scaffold N90 (Mb)	22.3	0.12
	Largest scaffold (Mb)	46.6	6.9
**Annotation**	Number of genes	40 420	31 007
	Repeat content (%)	41.7	52.9
	Number of ncRNAs	2555	NA
	BUSCO (%)	98.1%	94%
	GC content (%)	43.9	33.7

We next performed genome annotations using the *BRAKER2* pipeline, combining evidence from *ab initio* prediction, protein homologs, and multi-tissue (root, shoot, leaf, and fruit) transcriptome sequencing data. The genome annotation pipeline predicted a total of 40 420 protein-coding genes and 2555 non-coding RNAs for *D. longan* (Table 1). The longan genome has an overall guanine-cytosine (GC) content of 34% and a gene density of 89 genes per Mb (Supplementary Table 2). About 89.0% of the genes were annotated with the non-redundant protein sequence database (Nr), and 84.6% of the genes were annotated with Kyoto Encyclopedia of Genes and Genomes (KEGG) terms (Supplementary Table 3). Repetitive elements make up 41.7% of the *D. longan* genome, 54.9% and 25.4% of which are long terminal repeat retrotransposons (LTRs) and DNA transposons, respectively. Two major LTR subtypes, LTR-*Copia* (179.64 Mb) and LTR-*Gypsy* (66.18 Mb), represent 8.55% and 15.53% of the longan genome, respectively (Supplementary Table 4).

### Comparative genomics and synteny analysis

**Figure 2:**
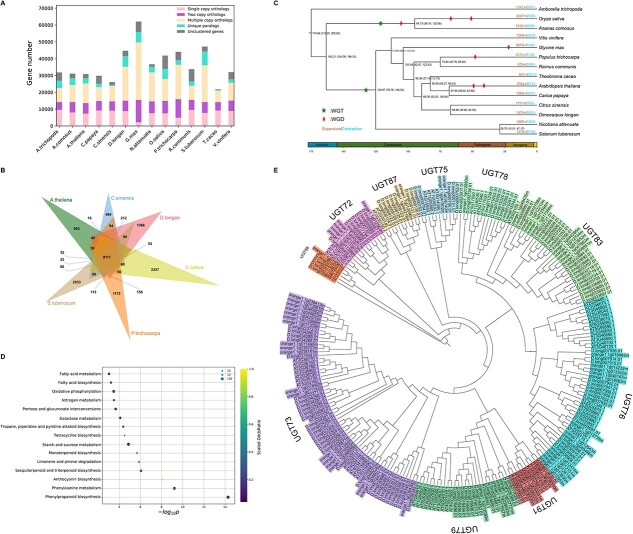
**Phylogenomic genomics of *Dimocarpus longan.*** (**A**) Summary of gene family clustering of *D. longan* and 13 related species. Single copy orthologs: 1-copy genes in an ortholog group. Multiple copy orthologs: multiple genes in an ortholog group. Unique orthologs: species-specific genes. Other orthologs: the rest of the clustered genes. Unclustered genes: number of genes outside of cluster. (**B**) Comparison of orthogroups (gene families) among six angiosperm species, *D. longan* (longan), *A. thaliana* (*Arabidopsis*), *C. sinensis* (citrus), *S. tuberosum* (potato), *P. trichocarpa* (poplar), and *O. sativa* (rice). (**C**) Phylogenetic relationships and divergence time estimates (with confidence intervals). The numbers of gene family expansions and contractions are indicated by red and blue numbers, respectively. (**D**) Bubble plot summarizing the most significantly enriched KEGG terms associated with expanded gene families in *D. longan*. The x-axis is the log10 transformed p-value. The size of the bubble is scaled to the number of genes. The color scale represents the scale of odds ratio in observed versus expected (genomic background) numbers of genes annotated with specific KEGG terms. (**E**) A phylogenetic tree of UGTs (UDP-glucosyltransferases) in three angiosperms, including *D. longan*.

Next, we performed intraspecies synteny analysis of the *D. longan* genome to investigate its genome evolutionary history. Intraspecies syntenic gene pairs in *D. longan* were identified using *MCScanX,* which supported the presence of a whole genome triplication (WGT) event in the longan genome (Supplementary Figure 2A). The synonymous substitution rate (*Ks*) distribution for syntenic gene pairs also suggested that the *D. longan* genome had experienced the WGT (Supplementary Figure 2B). In addition, the 1:1 ratio of syntenic blocks between longan and grape (*Vitis vinifera*) and the 1:2 ratio of syntenic blocks between longan and poplar (*Populus trichocarpa*) indicated that longan had only the WGT (γ) event and did not have other whole genome duplications (WGDs) (Supplementary Figure 2C). To reveal the genome evolution and divergence of longan, we performed phylogenomic analysis of longan and thirteen representative plant species, including eight Rosids (*Citrus sinensis, Carica papaya, Arabidopsis thaliana*, *Theobroma cacao, P. trichocarpa, Ricinus communis, Glycine max, V. vinifera*)*,* two Solanaceae (*Solanum tuberosum, Nicotiana attenuata*)*,* one Poaceae (*Oryza sativa*), and a basal angiosperm (*Amborella trichopoda*)*.* Orthogroup (gene family) identification revealed that these plants shared 7530 orthogroups, 137 of which are single-copy groups (Figure 2A; Supplementary Table 5). In particular, we identified 1366 orthogroups unique to *D. longan* compared to *A. thaliana*, *C. sinensis*, *S. tuberosum*, and *P. trichocarpa* (Figure 2B). The multiple sequence alignments of 137 single-copy orthologs in 14 species were concatenated and used for phylogeny construction and divergence time estimation using *MCMCTree* calibrated with fossil records (Figure 2C). Among the thirteen species, longan was phylogenetically closest to *C. sinensis*. The two species shared a last common ancestor at around 67 million years ago (Mya) that diverged from the asterids (*N. attenuata, S. tuberosum*) at around 125 Mya (Figure 2C).

### Expanded gene families related to phenylpropanoid biosynthesis and UDP-glucosyltransferases

Gene family contraction and expansion are the evolutionary forces that drive rapid speciation and result in the diversification of plants [9]. Gene family analysis suggested that the longan genome has 1474 expanded and 2424 contracted gene families (Figure 2C) compared with the common ancestor of *C. sinensis* and *D. longan*. KEGG enrichment showed that the expanded gene families were significantly enriched (*P < 0.05*) in “phenylpropanoid biosynthesis”, “phenylalanine metabolism”, “anthocyanin”, “sesquiterpenoid and triterpenoid biosynthesis”, and “monoterpenoid biosynthesis” (Figure 2D). The 97 expanded longan phenylpropanoid biosynthesis genes were classified into seven gene families: phenylalanine ammonia-lyase (PAL, 5 members), peroxidase (POD, 38 members), O-methyltransferase (OMT, 3 members), glycosyl hydrolase family 1 (GH1, 26 members), aldehyde dehydrogenase family (ADH, 18 members), AMP-binding enzyme (4 members), and beta-galactosidase (BGL, 3 members) (Supplementary Table 6), probably involved in the biosynthesis of various lignin precursors. Lignin is a major component of some plant cell walls and is likely to have been involved in longan speciation [19]. Structural lignins provide physical barriers against pathogen infection and mechanical support for plant growth and the long-distance transport of water and nutrients [20]. Key enzymes such as PAL, POD, and PPO of the phenylpropanoid and lignin pathways are involved in these processes. Catalyzing the first step in the phenylpropanoid biosynthetic pathway, PALs were expressed at higher levels in roots, leaves, and stems but not in green fruits (Supplementary Figure 3), consistent with a previous report [9]. In addition, 28 of the 38 PODs showed differential expression in four major tissues (leaves, stems, roots, and fruits) (Supplementary Figure 4). The phenylpropanoid pathway is a conserved biosynthetic pathway in plants that leads to the production of various phenylpropanoid secondary metabolites such as phenolics, lignins, and flavonoids. The longan expansion of genes encoding these biosynthetic enzymes suggests that this pathway has probably evolved to generate diverse types of phenylpropanoids in longan through natural and artificial selection. A previous transcriptome-based study contrasted with our findings, suggesting that structural genes in phenylpropanoid pathways showed contraction instead of expansion [9]. However, we argue that a high-quality genome assembly and annotation is essential for the correct and complete characterization of gene families, producing results that are more trustworthy than transcriptome analysis alone.

Plants have evolved exquisite mechanisms for the biosynthesis of phenylpropanoids through acylation, methylation, glycosylation, and hydroxylation [21]. Most of the compounds synthesized by the phenylpropanoid pathway can be glycosylated by UDP-glucosyltransferases (UGTs). UGTs are key glycosylation enzymes that stabilize and enhance the solubility of small metabolites to maintain intracellular homeostasis [22]. For example, UGTs glycosylate volatile benzenoids/phenylpropanoids, the monoterpene linalool, and a strawberry aroma 4-hydroxy-2,5-di-methyl-3(2H)-furanone. InterPro (IPR) protein domain enrichment analysis showed that the expanded longan gene families are significantly enriched in IPR domains such as UGTs and cytochrome P450s (Supplementary Figure 5). The longan genome encodes more (215) UGTs (Supplementary Table 7) than those of *Arabidopsis* (107), *C. sinensis* (135), *C. grandis* (145), and *V. vinifera* (181) but fewer than that of apple (241) [ 23]. UGTs also participate in plant development, growth, and defense responses. Phenylpropanoid metabolism plays important roles in resistance to pathogen infection through the UGTs^21^. A phylogenetic tree was constructed using UGT protein sequences from longan and other plants, including *Arabidopsis* and *Citrus* (Figure 2E), and 115 expanded UGTs were divided into ten groups. Five groups (A, D, E, G, and L) expanded more than the others, although the numbers of genes in these groups varied widely among species [23]. In longan, groups A, D, H, I, and L expanded more than the other groups. For example, the numbers of longan UGTs in group D (31 UGTs, UGT73) and group I (19 UGTs, UGT83) were significantly increased compared with those in *Arabidopsis* and *Citrus*. A group D member, UGT73C7, mediated the redirection of phenylpropanoid metabolism to hydroxycinnamic acids (HCAs) and coumarin biosynthesis under biotic stress, resulting in SNC1-dependent *Arabidopsis* immunity [24]. In group I, the number of UGTs was highest compared with other fruits such as peach (5 UGTs), apple (11 UGTs), and grapevine (14 UGTs). UGT83A1 (GSA1) was required for metabolite reprogramming under abiotic stress through the redirection of metabolic flux from lignin biosynthesis to flavonoid biosynthesis and the accumulation of flavonoid glycosides, which coordinately confer high crop productivity and enhanced abiotic stress tolerance [25]. A total of 115 expanded UGT genes were unevenly distributed on the 15 chromosomes of the longan genome (Supplementary Figure 6). Chromosome 15 contained the most UGT genes (24), followed by 20 on Chromosome 13. Many of these UGT genes form clusters on several chromosomes (Supplementary Figure 6), suggesting that they are probably derived from tandem duplications of ancient UGT genes during longan evolution. Transcriptomic profiling suggested that 96 UGTs were differentially expressed in longan, with four (accounting for 4.2%), fourteen (14.6%), and ten (10.4%) UGTs uniquely expressed in leaves, roots, and fruit, respectively (Supplementary Figure 7, Supplementary Table 8).

### Population structure, migration, and genetic admixture of longan cultivars

**Figure 3:**
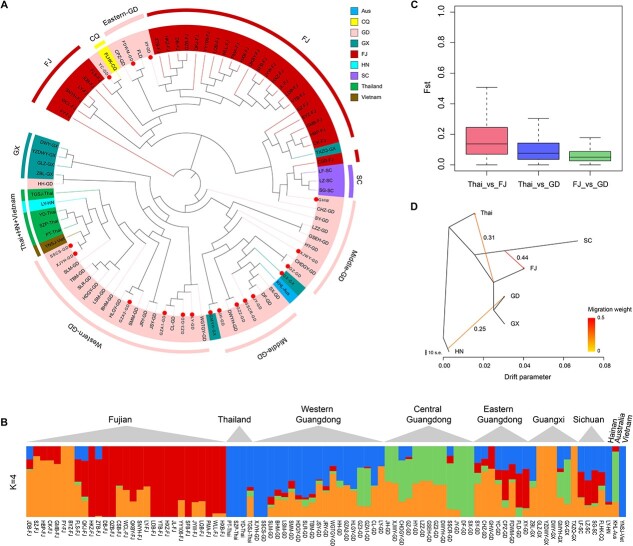
**Population structure and admixture analysis of *Dimocarpus longan*.** (**A**) A neighbor-joining phylogenetic tree of all *D. longan* individuals was constructed using SNPs. The artificial breeding individual was marked with red dots inside. Colors represent different geographic groups. (**B**) A biogeographical ancestry (admixture) analysis of *D. longan* accessions with four ancestral clusters colored differently in the heatmap, in which each column represents a longan sample. (**C**) Distribution of *Fst* values (a measure of genetic differentiation) between longan populations from Thailand (Thai), Fujian (FJ), and Guangdong (GD). (**D**) Maximum-likelihood tree and migration events among seven groups of *D. longan*. The migration events are colored according to their weight.

To understand longan genomic dynamics across its current distribution range in southern China and southeast Asian countries, we performed whole-genome resequencing analysis of 87 accessions (Supplementary Table 9) from five southern provinces in China (Guangdong, Fujian, Guangxi, Sichuan, and Hainan) and three other countries (Thailand, Vietnam, and Australia) with an average sequencing depth of 50×. Read mapping to the longan reference genome and variant detection yielded 29 730 132 single nucleotide polymorphisms (SNPs). After filtering, 11 421 213 high-quality SNP loci (minor allele frequency > 5%) were used for subsequent population genetic analyses. Although Guangdong borders on Fujian, the climate of the two provinces differs greatly during the longan growing season. After generations of planting and screening, different cultivation areas have formed their own longan variety characteristics and types. Using the genetic variant data, we analyzed the population structure within these longan cultivars using phylogenomic analysis and principal component analysis. Phylogenomic analysis clustered 87 longan accessions into relatively distinct domestic Guangdong and Fujian groups after the removal of artificial breeding populations (Figure 3A). Three Sichuan cultivars were next to the Fujian group and distant from the Guangdong group. Notably, two Guangdong cultivars, FLD and CPZ, were clustered with the Fujian group, probably because they come from eastern Guangdong, adjacent to Fujian. Guangdong cultivars are divided into two subgroups, the “Shixai” (SX)-centered group and the “Chuliang” (CL)-centered group from central and western Guangdong, respectively (Figure 3A); these are also the two main cultivars widely grown in Guangdong and Guangxi. Consistent with the phylogenetic tree, the principal component analysis showed that the Guangdong and Fujian cultivars were grouped separately overall, and the Thai and Vietnamese populations were distant from the Chinese populations if artificial breeding cultivars were removed (Supplementary Figure 8).

To investigate the genetic background of longan from various regions, we performed a biogeographical ancestry (admixture) analysis based on high-quality SNPs and tested it with ancestral group values (k) ranging from 2 to 8. With a choice of four ancestral groups (k = 4), which gave the smallest cross-validation error (Supplementary Figure 9), the admixture analysis revealed a distinct genetic structure within longan accessions of different geographic origins. Longan cultivars from Fujian are primarily composed of two ancestral groups, whereas Guangdong, Guangxi, and Sichuan cultivars contain fractions of all four ancestral groups, indicating their more complex ancestral backgrounds (Figure 3B). The more similar ancestry composition between eastern Guangdong and Fujian cultivars is consistent with the geographic closeness of the two growing regions, suggesting a common ancestral origin or a possible exchange of cultivars between the two regions. By contrast, Thai and Vietnamese cultivars have a simple composition overall, with one predominant ancestral group, most likely shared with western Guangdong (Figure 3B). Thai cultivars were genetically more related to western Guangdong cultivars (Figure 3A) but distant from Fujian cultivars. Consistent with this result, we also detected stronger genetic differentiation (measured as the *Fst* value) between Thailand and Fujian than between Thailand and Guangdong (Figure 3C). Notably, the Australian cultivar has a genetic background that resembles the middle Guangdong cultivar, suggesting that it is a possible cultivar of middle-Guangdong origin that has recently been introduced into Australia.

With the diverse ancestral backgrounds of these longan cultivars, we were curious about the migration history of longan germplasms and therefore investigated potential gene flows among different growing areas due to such migration using TreeMix analysis. Given its reported origin in China, many wild longan resources are present in the Yunnan and Hainan provinces of China [6]. Therefore, the Hainan cultivar was used as an outgroup in this analysis. The TreeMix analysis detected a migration event directed from Hainan to Guangdong. The highest gene flow (migration weight 0.44) was observed between Sichuan and Fujian (Figure 3D). Gene flows were also detected from the Fujian, Guangdong, and Guangxi populations to Thailand (migration weight 0.31) (Figure 3D). The detection of gene flows was consistent with historical records of longan migration. Longan was first cultivated in the “Ling-nan” district of China, including Guangdong, Guangxi, and Hainan, about 2000 years ago, recorded by the painting of “San Fu Huang”. According to historical records, longan was not successfully moved to northern China-Shaanxi Province but was successfully introduced to Sichuan and then to Fujian, with suitable climate conditions (Yang Fu, “Chronicles of the South”, 1st century A.D.). Taken together, our analysis results broadly matched historical records that there was gene flow from Hainan wild germplasms to Guangdong, then a strong flow from Sichuan to Fujian, and finally gene flow from China to Thailand.

### GWAS for longan fruit quality traits

We next performed GWAS for fruit qualities to uncover QTLs underlying these traits. Phenotyping data, including pericarp thickness, pulp thickness, fruit horizontal diameter, total soluble solids, edible rate, and seed weight, were collected from 80 longan cultivars at the Longan Germplasm Repository of Guangdong Province that were genotyped in this study. To validate the reliability of the phenotype data, we calculated the Pearson correlation coefficient. Consistent with a previous study of longan [26], the coefficient of variation of single fruit weight and seed weight was the largest, whereas the coefficient of variation of fruit weight, pericarp thickness, and edible rate was the smallest (Supplementary Figure 10). GWAS analysis using 11 421 213 biallelic SNPs identified ten candidate QTLs that were significantly associated with six longan traits (p-value <1e−6) (Supplementary Table 11), associating 12 503, 7834, 11 184, 6348, 46 182, and 23 523 SNPs with pericarp thickness, pulp thickness, fruit horizontal diameter, edible rate, seed weight, and total soluble solids, respectively (Supplementary Figure 11). Genomic regions associated with seed weight contained several genes (Supplementary Table 9) related to seed development, such as *UBQ10*, *UBIQ1*, *THI4*, *CBL10*, and *CTPS2*. Specifically, a genomic region 0.94–0.99 Mb on Chromosome 6 contained three tandemly duplicated *DlBGL42* genes encoding β–glucosidase (Supplementary Table 12), consistent with reports that seed size is controlled by *Clbg1* (watermelon β–glucosidase) via decreased ABA content [27].

**Figure 4:**
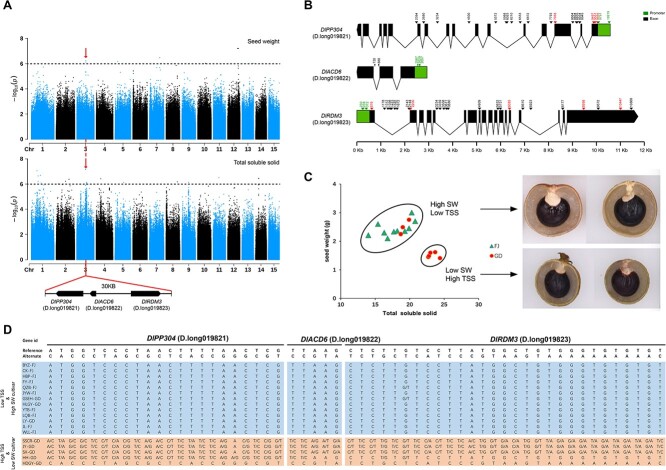
**GWAS mapping of seed weight and total soluble solids in longan fruit.** (**A**) Top: Manhattan plot summarizing the GWAS results for seed weight based on analysis performed with a randomly down-sampled SNP call set. Middle: Manhattan plot of GWAS results for total soluble solids. Dotted lines represent the Bonferroni significance threshold. Red vertical lines represent the overlapping region of the two traits as highlighted below. Bottom: close-up of highlighted regions with three genes in the vicinity of the GWAS peak; (**B**) Diagram of three genes with respect to reference sequences and the haplotypes observed in samples collected from China. Black solid arrows indicate synonymous SNPs, and red solid arrows indicate non-synonymous SNPs specific to varieties with high total soluble solids (TSS) and low SW (seed weight); (**C**) Correlations of SW and TSS; (**D**) SNPs within the three genes for the most extreme TSS and SW phenotypes at each end of the high-type and low-type distributions (orange mutation is heterozygous to homozygous; blue: non-mutated) (see Supplementary Table 11 for all samples).

Fruit sweetness is generally measured by total soluble solids content [28]. For a long time, high total soluble solids and edible rate have been key longan traits selected in longan breeding [14]. Interestingly, GWAS identified a genomic region located on Chromosome 3 (at ~18.37–18.45 Mb) that harbored QTLs shared between total soluble solids and seed weight (Figure 4A). A SNP variance analysis of this region found polymorphic site mutations of ~37 kb at approximately 18.379 to 18.416 Mb, harboring the three successive genes *DlPP304* (*D. long019821.01*), *DlACD6* (*D. long019822.01*), and *DlRDM3* (*D. long019823.01*), which encode a mitochondrial pentatricopeptide repeat-containing protein, an accelerated cell death protein, and an RNA-directed DNA methylation protein, respectively (Figure 4B). The co-localization of trait-linked loci usually reflects significant positive correlations among different traits [29]. Sugar content is a fruit characteristic that varies among cultivars and contributes to the distinctive flavor profile of longan varieties. There are reports that sugar contents are affected by fruit size and seed weight in sapodilla [30], and soluble sugar accumulation affects *Arabidopsis* seed size [31].

We next analyzed seed weight and total soluble solids phenotypes in 80 longan cultivars (Supplementary Figure 12). We observed that a group of ten Fujian and three Guangdong varieties displayed low soluble solids but bigger and heavier seeds, whereas another group of five Guangdong varieties had high soluble solids but smaller and lighter seeds (Figure 4C; Supplementary Figure 10). Examination of SNPs in the *DlPP304*, *DlACD6*, and *DlRDM3* regions suggested that varieties with high soluble solids and low seed weight contained a larger number of variants than those with low soluble solids and high seed weight (Figure 4D, Supplementary Table 11). In particular, we found that the HDGY-GD variety, which contained mostly homozygous variants at *DlPP304*, *DlACD6*, and *DlRDM3* gene regions, had the highest total soluble solids and the lowest seed weight among the cultivars (Figure 4D). Interestingly, most of the SNPs within the three genes were heterozygous in the group with high total soluble solids, hinting that sugar content is likely to be associated with heterosis, as high total soluble solids has long been a desired trait in longan breeding programs through hybridization. Previous studies have found a large proportion of the yield advantage of hybrids over their parents of elite inbred varieties [32, 33]. For most of the heterosis-related loci identified, dominance or incomplete dominance of heterozygous loci plays an important role. However, this hypothesis remains to be validated in further experiments. In summary, our GWAS analysis revealed a list of QTLs and candidate genes for further validation by functional genomics or forward genetics approaches.

## Discussion

Longan is one of the most economically important fruit trees in China and Southeast Asia. However, molecular breeding of longan cultivars has been slow because of a lack of genomic and biotechnological resources. Using a combination of single-molecule (PacBio) and Hi-C sequencing data, we successfully produced a chromosome-level longan genome assembly, followed by protein-coding gene annotations. This genome assembly for longan represents a significant improvement over a current draft reference genome based on short-read sequencing data [9]. The improvement mainly stems from assembly of contigs using PacBio long reads, followed by scaffolding based on chromatin interaction information given by Hi-C data. As longan is primarily an outbreeding tree species, it typically has a high heterozygosity rate, which presents a challenge to the contiguous assembly of its diploid genome. The JDB cultivar used for *de novo* genome assembly has a relatively low heterozygosity rate of 0.3%, probably due to repeated inbreeding. Together, these two factors facilitated the highly contiguous assembly of the genome. The repetitive content (41%) in our genome assembly is less than that reported previously for an assembly (52%) based on Illumina short reads by Lin et al. [9], indicating that long read sequencing has helped to resolve the ambiguous placement and assembly of repetitive regions that are typically prone to mis-assembly from short reads. The high BUSCO score (98%) shows that completeness of the present longan assembly is also improved compared with that of Lin et al. [9], although we admit that there is still much to do to obtain a complete longan genome sequence in the future. In short, we present a high-quality, chromosome-scale genome assembly of longan, the best longan genome assembly reported to date.

Using this reference genome, we investigated the evolution of the longan genome and showed that it shared a whole genome triplication and lacked additional WGDs. Lin et al. [9] performed 4DTv analysis of their longan genome assembly and found that longan had an ancient genome duplication event, but no information was given with respect to whether the event occurred in related plants. By contrast, we conducted inter-species analysis using the longan and grape genomes and showed that longan and grape had a 1:1 syntenic relationship, confirming that the ancient duplication event in longan [9] was actually a whole genome triplication (γ event) shared with grape and other core eudicots. Given that the poplar genome underwent a post-γ WGD, the 1:1 and 1:2 syntenic relationships between longan and grape and between longan and poplar suggested the lack of a WGD following the divergence of grape and longan and of longan and poplar. Both Lin et al. [9] and our study identified a peak in *Ks* or 4DTv distribution for longan paralogs, indicating that many gene duplications have occurred recently in the longan genome, probably resulting from the activities of transposable elements.

The delightful taste and rich nutrition of longan fruits are among many unique traits of this plant. Longan fruit are rich in polyphenolic compounds. Genomic analysis of longan can reveal critical information about how the plant evolved such a unique chemical reservoir. We detected significant gene family expansions in biosynthetic genes of phenylpropanoids, terpenoids (sesquiterpenoids and triterpenoids), etc., compared with related plant lineages, suggesting that the longan genome has evolved towards innovation of these secondary metabolites through natural or artificial selection. By contrast, Lin et al. [9] analyzed their draft longan genome assembly and also showed that the longan genome had experienced gene family expansions and contractions. However, no functional terms related to specialized metabolism were found to be enriched in the expanded or contracted gene families they identified, although they did show enrichment of terms related to general biological processes such as cellular component organization. These different enrichment results may be explained by several factors, such as an improved genome assembly and annotations, the selection of plant genomes for comparison, and the functional enrichment analysis tools used. Our analysis also identified the expansion of UGT gene families, which are essential for the diversification of structural features and control the final oxidation, hydroxylation, and glycosylation steps to yield secondary metabolites in longan. The exact roles of UGTs in the formation and modification of specific longan natural products that give longan fruits their unique taste and nutrition remain to be determined.

Longan is widely grown in China and many Southeast Asian countries where longan cultivars were initially introduced from China, the origin of longan with the largest cultivation area in the world. Chinese longan consists mainly of the Guangdong population, the Fujian population, the Guangxi population, and so forth. Exchange of longan germplasms among different growing regions probably led to genetic introgression. Because longan can undergo both inbreeding and outbreeding in the natural environment, its genetic background and population structure are rather complex and await elucidation. The 87 cultivated varieties in this study were mostly collected from mainland China and a few neighboring South Asian countries; they have excellent characteristics and are often used as breeding parents, but most evolved through long-term natural hybridization. The evolutionary model of natural hybridization is unclear, resulting in the unclear genetic background of germplasm resources. As a result, confusion among names of longan varieties is not uncommon, such that different varieties have the same name, or the same variety has different names. Combining a high-quality genome assembly and population genomic sequencing of longan varieties, we have resolved the complex genetic background of longan, revealed its population admixture model, and deduced possible migration routes of longan consistent with historical records.

The high-quality SNP molecular markers generated in this study enabled the discovery of multiple QTLs strongly associated with useful traits such as seed weight and soluble solids, providing excellent resources for future molecular breeding. A QTL locus containing the three successive genes *DIPP304*, *DIACD6*, and *DIRDM3* was significantly associated with total soluble solids and seed weight. Literature shows that the homologs of these genes have been reported to function in sugar content and fruit ripening, seed set, fruit abscission, and so forth [34–37]. The PPR (pentatricopeptide repeat) gene affecting sugar content in plants is likely to be involved in regulating cellular carbon metabolism. For example, the mitochondrial PPR protein SLO2 is required for carbon energy balance in *Arabidopsis*, and sugar contents were much lower in the *slo2* mutants [34]. Thus, the longan *DlPP304* gene encoding a PPR gene homolog probably plays similar roles in regulating the carbon energy balance that affects soluble solid contents in fruits. As for DlACD6, its *Arabidopsis* homolog AtACD6 (ACCELERATED CELL DEATH 6) is a transmembrane ankyrin repeat protein that modulates the activity of pattern recognition receptors (PRRs) associated with hybrid necrosis [38]. A single amino acid insertion of ACD6 was sufficient to convert the reference Col-0 allele into a necrosis-inducing allele [36], resulting in reduced seed set. Therefore, based on the literature and GWAS results in our study, we speculate that genetic variation in *DlACD6* probably contributes to variable seed characteristics such as seed weight among longan cultivars. DlRDM3 is a protein homolog of the RNA-directed DNA methylation (RDM) family. Repression of the RDM pathway is reportedly required for fruit abscission in *C. sinensis* [37]. Taken together, these findings pave the way for research through the integration of genomic, transcriptomic, and metabolomic data to provide abundant promising candidates for future improvement of desired longan traits.

## Materials and methods

### Germplasm genetic resources

A 30-year-old *D. longan* tree cultivar named JDB from the Longan Germplasm Repository of Guangdong Province (which belongs to the Institute of Fruit Tree Research at the Guangdong Academy of Agricultural Sciences in China) was used for genome sequencing and *de novo* assembly. Eighty-six additional *D. longan* cultivars (Supplementary Table 9) from the Longan Germplasm Repository of Guangdong Province were collected for genome resequencing.

### DNA and RNA isolation

The longan cultivar JDB was planted in the Longan Germplasm Repository of Guangdong Province. Fresh and healthy young leaves were collected, cleaned, and used for genomic DNA isolation and sequencing. Genomic DNA was extracted from young fresh leaves of *D. longan* using the modified cetyltrimethylammonium bromide (CTAB) method [39]. The concentration and purity of the extracted DNA were assessed using a Nanodrop 2000 spectrophotometer (Thermo, MA, USA) and a Qubit 3.0 system (Thermo, CA, USA), and the integrity of the DNA was measured using pulsed-field electrophoresis with 0.8% agarose gel. In addition, fresh leaves and other tissues (roots, shoots, and young fruits) of the JDB cultivar were collected for RNA isolation and transcriptome sequencing. Total RNA was isolated with the RNAprep Pure Plant Kit (Tiangen Biotech) according to the manufacturer’s instructions. The integrity and quantity of extracted RNA were analyzed on an Agilent 2100 Bioanalyzer. For each tissue, three biological replicates were prepared for sequencing.

### Genome and transcriptome sequencing

DNA sequencing libraries were constructed and sequenced on the Illumina NovaSeq 6000 platform at 50× depth according to the manufacturer’s protocols (Illumina). To generate long-read sequencing reads for *D. longan*, DNA libraries were prepared for PacBio SMRT sequencing following the PacBio standard protocols and sequenced on the Sequel platform. In brief, genomic DNA was randomly sheared to an average size of 20 kb using a g-Tube (Covaris). The sheared gDNA was end-repaired using polishing enzymes. After purification, a 20-kb insert SMRTbell library was constructed according to the PacBio standard protocol with the BluePippin size-selection system (Sage Science), and sequences were generated on the PacBio Sequel (9 cells) and PacBio RS II (1 cell) platforms by Biomarker Technologies. Raw subreads were filtered based on read quality (≥0.8) and read length (≥1000 bp). For chromosome-level genome scaffolding, Hi-C libraries were prepared from fresh leaves and sequenced on the Illumina HiSeq X Ten platform. DNA was digested with HindIII enzyme, and the ligated DNA was sheared into sizes of 200–400 bp. The resulting libraries were sequenced using the Illumina NovaSeq 6000 platform. For transcriptome sequencing, RNA sequencing (RNA-seq) libraries were constructed using the True-Seq kit (Illumina, CA) and sequenced using the Illumina HiSeq X Ten platform. Illumina raw reads were trimmed using *Trimmomatic* (v0.39) [40] with parameters “LEADING: 10 TRAILING:10 SLIDINGWINDOW:3:20 MINLEN:36” to remove adapter sequences and low-quality reads, yielding a total of ~77.7 Gb clean RNA-seq data from four tissues.

### Genome assembly and evaluation

To estimate the genome size and heterozygosity level of *D. longan*, cleaned Illumina PE reads were used for k-mer spectrum analysis with *GenomeScope* (v2.0) [41] based on 21-mer statistics. PacBio SMRT reads were used for *de novo* genome assembly with the *Canu* pipeline (V1.9) [15] using the parameters “correctedErrorRate=0.045 corMhapSensitivity=normal ‘batOptions=-dg 3 -db 3 -dr 1 -ca 500 -cp 50”. Alternative haplotig sequences were removed using *purge_*dups [42] with default settings, and only primary contigs were retained for downstream analysis. To correct base-pair-level errors in raw assembly sequences, two rounds of polishing were conducted using high-quality Illumina DNA reads with *Pilon* (v1.23) [16]. The longan contigs were further anchored to chromosomes using *3D-DNA* [17] based on the Hi-C contact map, followed by manual correction using *Juicebox* (v1.11.08) [43] to fix assembly errors. The completeness of the genome assembly was assessed by BUSCO v1.22^18^ using the 2121 eudicotyledons_odb10 single-copy genes. PacBio sequence reads and Illumina DNA reads were aligned to the genome sequences using minimap2 [44] and BWA [45], respectively.

### Repetitive element annotation

We used a combination of the *de novo* repeat library and homology-based strategies to identify repetitive structures. *TransposonPSI* [46] was used to identify transposable elements. The *GenomeTools* suite [47] (LTR harvest and LTR digest) was used to annotate LTR-RTs with protein HMMs from the Pfam database. Then, a *de novo* repeat library of the longan genome was built using *RepeatModeler* [48], and each of the three repeat libraries was classified with *Repeat_Classifier*. Subsequently, the non-redundant repeat library was analyzed using BLASTx to search the transposase database (evalue = 1e−10) and non-redundant plant protein database (evalue = 1e−10) to remove protein-coding genes. Then, the *de novo* repeat library was used to discover and mask the assembled genome with *RepeatMasker* [49] with the “-xsmall -excln” parameter.

### Prediction and annotation of protein-coding genes

For gene structure annotations, the RNA-seq data from four different tissues were aligned to the repeat-soft masked genome using *STAR* [50], which generates intron hints for gene structure annotation. The structural annotation of protein-coding genes was performed using *BRAKER2* [51] by combining the aligned results from *ab initio* predictions, homologous protein mapping, and RNA-seq mapping to produce the final gene prediction. Genes with protein length < 120 amino acids and expression level < 0.5 TPM were removed. Predicted genes were assigned functions by performing BLAST searches against the NCBI non-redundant protein database with an e-value threshold of 1e−10. In addition, a comprehensive annotation was also performed using *InterProScan* (5.36–75.0) [52].

### Comparative genomics analysis

Putative orthologs were identified using protein sequences from two monocots, ten eudicots, *Amborella trichopoda*, and longan. Only the longest protein sequence was selected as representative of each gene. Orthogroups were inferred with *OrthoFinder* (v2.4.1) [53]. The species tree was used as a starting tree to estimate species divergence times using *MCMCTree* in the *paml* package (v4.9) [54]. Speciation event dates for *Ananas comosus*-*O. sativa* (102–120 MYA), *P. trichocarpa*-*R. communis* (70–86 MYA), *A. thaliana*-*Carica papaya* (63–82 MYA), and *G. max*-*C. sinensis* (98–117 MYA) obtained from *TimeTree* (www.timetree.org) were used to calibrate the divergence time estimates. We conducted two independent *MCMCTree* runs using the following settings: burnin = 20 000, sampfreq = 30, and nsample = 20 000.

The ortholog count table and phylogenetic tree topology inferred from *OrthoFinder* were provided to *CAFÉ* (v4.2) [55], which identifies significant expansion or contraction in each gene family across species using a random birth and death model to estimate the size of each family at each ancestral node. Among expanded gene families, longan genes enriched with IPR002213 (UDP-glucuronosyl/UDP-glucosyltransferase) and IPR036396 (Cytochrome P450 superfamily) and their ortholog CDS sequences from the *A. thaliana* and *C. sinensis* genomes were retrieved. Multiple sequence alignment was performed using *MUSCLE* (v3.8.1551) [56] software. *IQ-TREE* was used to construct a maximum likelihood tree with parameters “-m MF”. The tree file was loaded into the Interactive Tree of Life (iTOL) web server for tree visualization and figure preparation [57].

### Transcriptomic analysis

After removing adapters and trimming low-quality bases, RNA-seq reads were mapped to the longan reference genome using *STAR* [50] with parameters “--alignIntronMax 6000 –align IntronMin 50” and then using the *RSEM* tool [58] for transcript quantification. Outliers among the individual experimental samples were verified based on the Pearson correlation coefficient (*r*^2^ ≥ 0.85). Differential expression analysis was performed using the *DESeq2* package [59]. Genes were differentially expressed between two conditions if the adjusted p-value was <0.01 and fold change was >1.

### Genetic variation detection

Population resequencing reads were mapped to the chromosome-level genome assembly of longan using *BWA* [45] with default parameters. Alignments for each sample were processed by removing duplicate reads using *SAMtools* [60]. The mpileup function in *SAMtools* was used to generate mpileup files for each sample. *BCFtools* [61] was used to identify SNPs and small indels. The SNPs were filtered with criteria of read mapping quality (MQ) > 40, minimum coverage > 10, base quality > 30, and genotype missing rate < 20% (of all samples). The high quality SNPs were used in further analysis.

### Population structure analysis

The vcf-format SNP sets were transformed into binary ped format using PLINK [62]. To estimate individual admixture assuming different numbers of clusters, the population structure and ancestry were investigated using *ADMIXTURE* [63] based on all SNPs. A linkage disequilibrium pruning step was performed with *PLINK* [62] using the parameters “--indep-pairwise 50 10 0.1”. We analyzed the number (K) of ancestral clusters ranging from 2 to 8 and found that the cross-validation error was smallest at K = 4. We also inferred a population-level phylogeny across all groups using the maximum likelihood approach implemented in *TreeMix* [64]. For this analysis, samples with a genotype missing rate > 20% were filtered out. SNPs with an imputation info score < 0.8, minor allele frequency (MAF) < 5%, and significant deviation (p < 10e–4) from Hardy–Weinberg equilibrium (HWE) were also removed.

### GWAS analysis

Phenotypic data from the *D. longan* cultivars in the Longan Germplasm Repository of Guangdong Province, including pericarp thickness, pulp thickness, fruit horizontal diameter, total soluble solids, edible rate, and seed weight, were measured in 2020 and 2021. Fifty mature fruits per cultivar were harvested for phenotypic measurement from five forty-year-old longan trees that had even canopy sizes and fruit loads. To ensure the statistical power of the analysis, we used SNPs with MAF > 5% to represent the genotyping information for each GWAS. GEMMA (version 0.98.1) was used to carry out the GWAS analyses with a mixed linear model (MLM) [65]. Furthermore, to minimize false positives in GWAS, population structure was taken into account using a kinship matrix that was estimated using *PLINK* [62]. The genome-wide significance threshold was set to 1e−6.

## Acknowledgement

This project is supported by the Key-Area Research and Development Program of Guangdong Province (2020B020220006) and the Guangdong Provincial Crops Germplasm Nursery Construction and Resources Collection, Preservation, Identification & Evaluation Foundation. In addition, LG is supported by the National Natural Science Foundation of China (31970317) and a faculty startup package from Peking University Institute of Advanced Agricultural Sciences. The authors would also like to thank anonymous reviewers for their comments and suggestions to improve this manuscript.

## Author contributions

Project design and oversight: LG, JL, and WQ; Sample collection and curation: DG and SH; Conducting experiment and data analysis: JW, ZL, and LG; Result interpretation: LG, JW, JL, BL, and WQ; Figure and table preparation: LG, JW, and LZ; Manuscript writing and revision: LG, JW, BL, and WQ; Funding: JL and LG; All authors have read and approved the final version of this manuscript.

## Data availability

The genome sequencing data, RNA-seq data, Hi-C data, and genome assembly of *D. longan* generated in this study have been deposited in the Genome Sequence Archive (GSA) database at the National Genomics Data Center of China National Center for Bioinformation under accession number CRA004281.

## Conflict of interest statement

The authors declare no conflict of interest.

## Supplementary data

Supplementary data is available at *Horticulture Research * online.

## Supplementary Material

Web_Material_uhac021
