# Supplementary material for: Genomic insights into longan evolution from a chromosome-level genome assembly and population genomics of longan accessions
Source: Hortic Res. 2022 Feb 19;9:uhac021. doi: 10.1093/hr/uhac021 (PMC9071379; doi:10.1093/hr/uhac021)
Supplement: Web_Material_uhac021 [file web_material_uhac021.zip › Supplementary Figure 1-12.pdf]

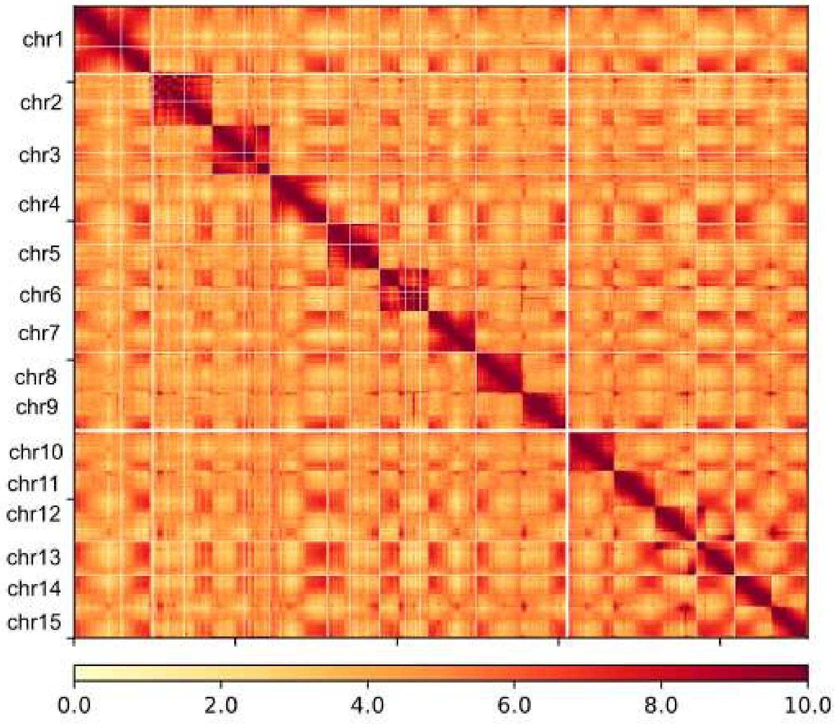

Supplementary Figure 1: Heat map of chromosomal interactions in the D. longan genome. chr1-chr15 represent the fifteen chromosomes in the D. longan genome. The horizontal and vertical coordinates represent the order of each 'bin' on the corresponding chromosome.

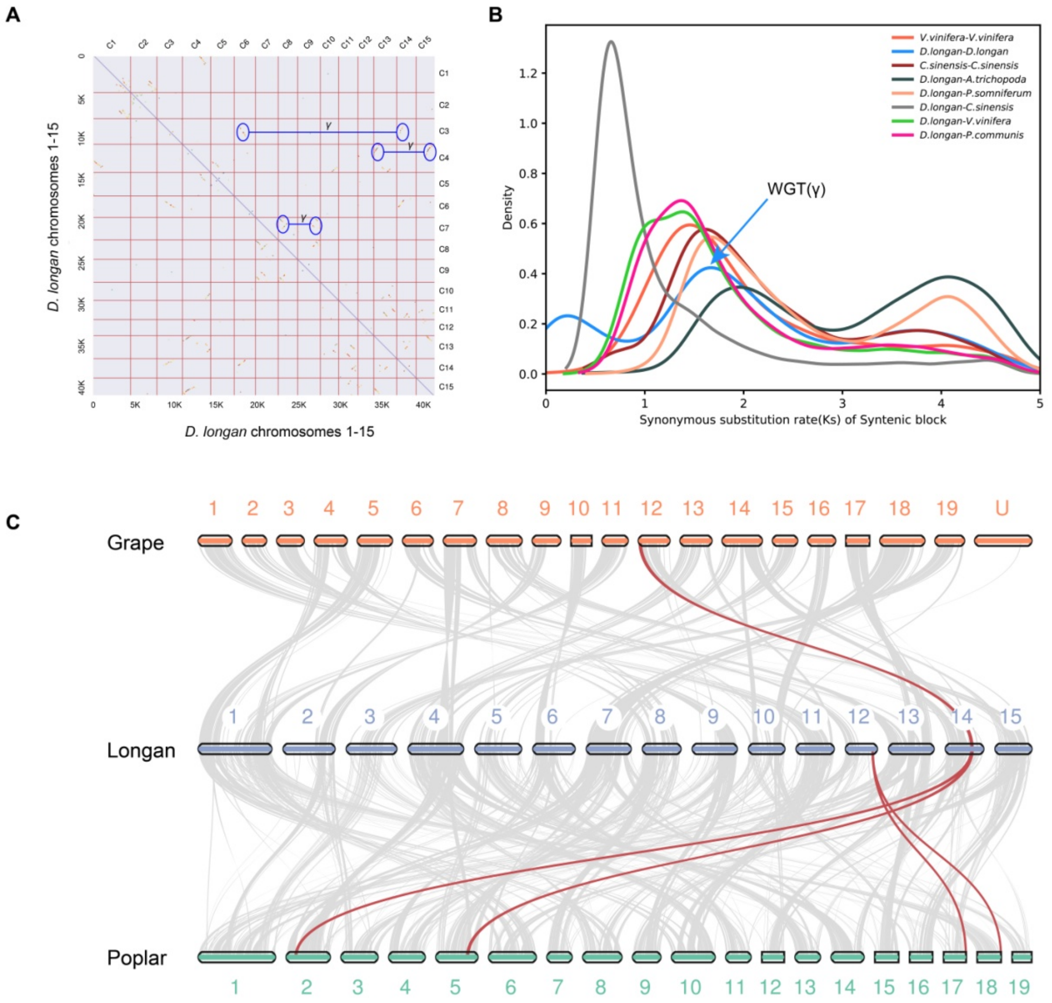

Supplementary Figure 2. Comparative genomics and synteny analysis of *Dimocarpus longan*. (**A)** Whole genome dot plot of *D. longan* showing intraspecies genome synteny based on syntenic gene pairs. The pair of black circles connected by a straight line highlight the syntenic blocks detected in *D. longan* genome, which corresponds to the whole genome triplication (γ event). (**B**) Distribution of *Ks* (synonymous substitution rate) density for syntenic paralogs or orthologs detected in pairwise comparisons among various plant genomes. **(C)** Karyotype macrosynteny plots displaying the collinear relationships for different chromosomes among grape (*Vitis vinifera*), longan (*Dimocarpus longan*) and poplar (*Populus trichocarpa*). The colored lines highlight the syntenic blocks conserved among three species.

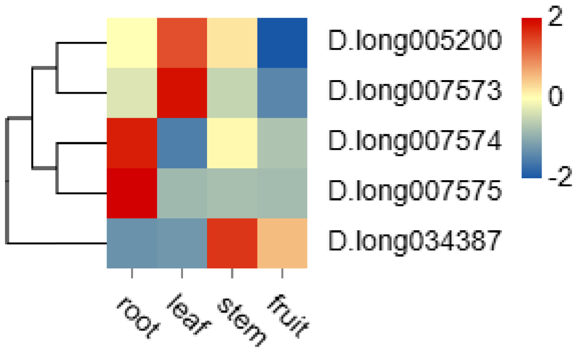

Supplementary Figure 3: The heatmap of phenylalanine ammonia-lyase genes (PALs) expressed in various longan tissues.

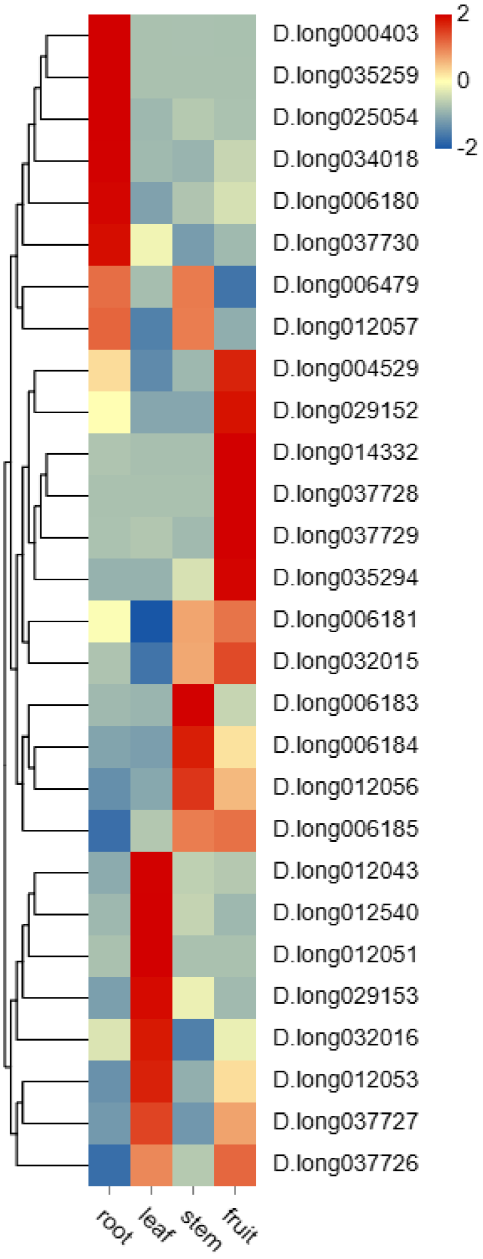

Supplementary Figure 4: The heatmap of peroxidase genes (PODs) expressed in various *Dimocarpus longan* tissues.

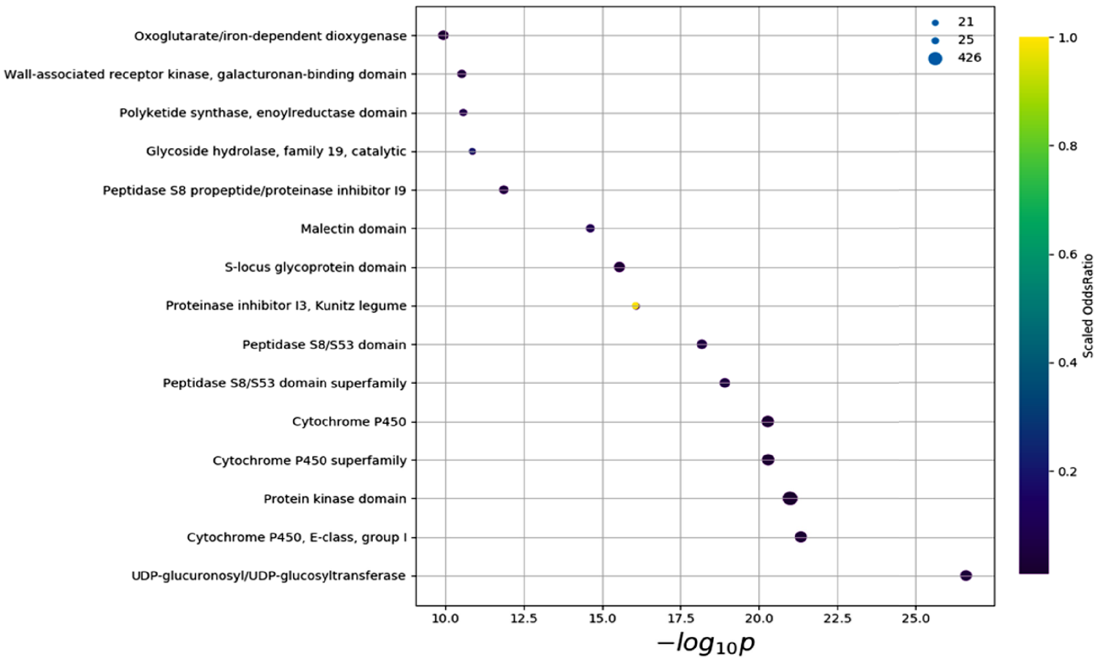

Supplementary Figure 5: InterPro protein domain enrichment analysis of *Dimocarpus longan* expanded gene families.

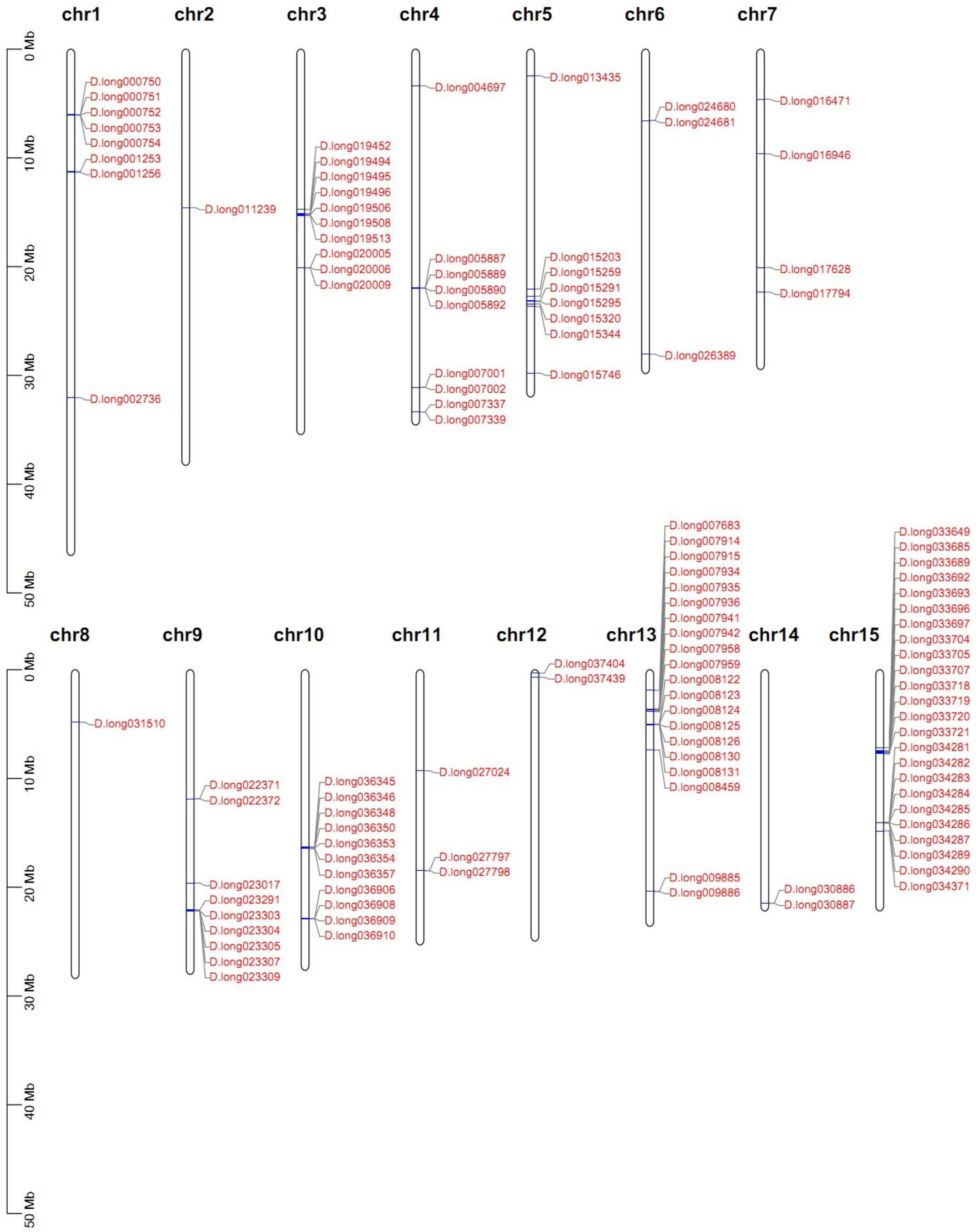

Supplementary Figure 6: Chromosome distribution of expanded longan UGTs. The longan UGTs were distributed among 15 chromosomes (chr1-chr15). Chromosome numbers are displayed at the top of each chromosome.

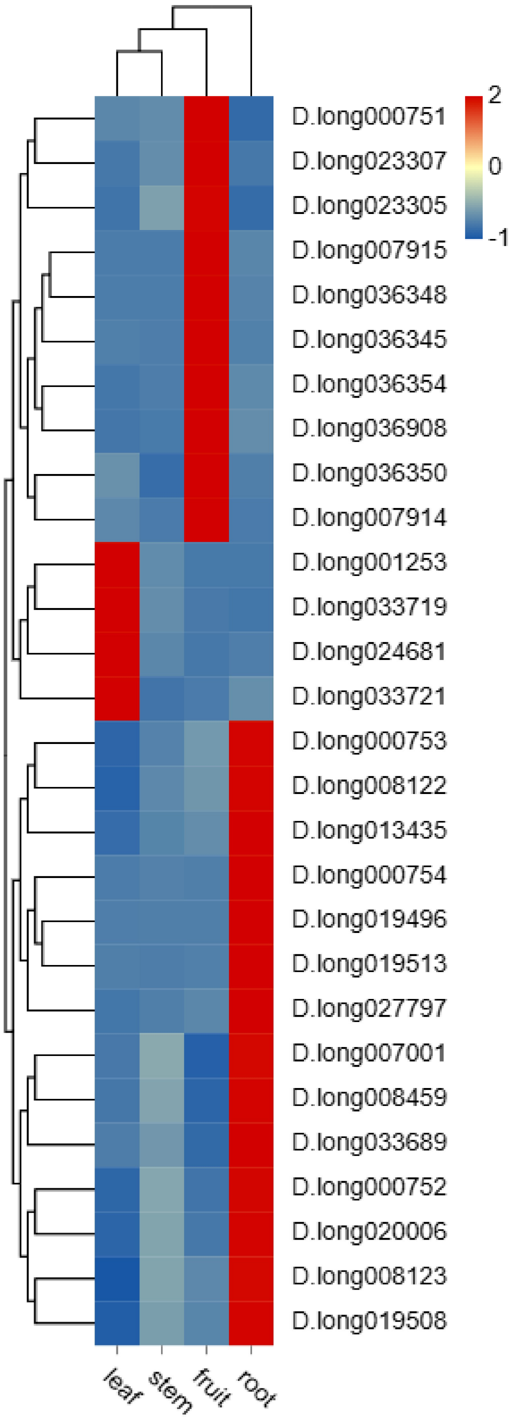

Supplementary Figure 7: The heatmap of UGTs genes expressed in various longan tissues.

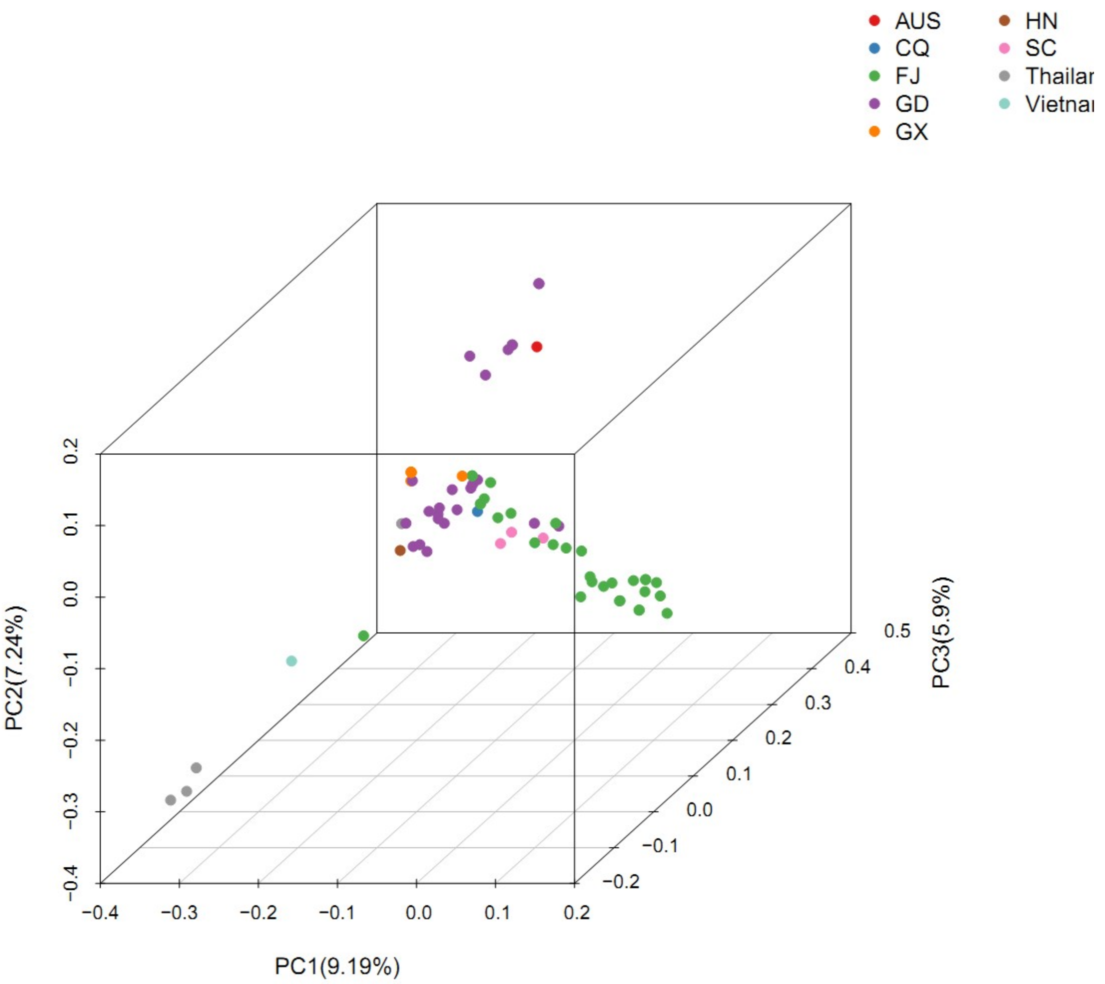

Supplementary Figure 8: Principle component analysis of *Dimocarpus longan* samples based on genotypes.

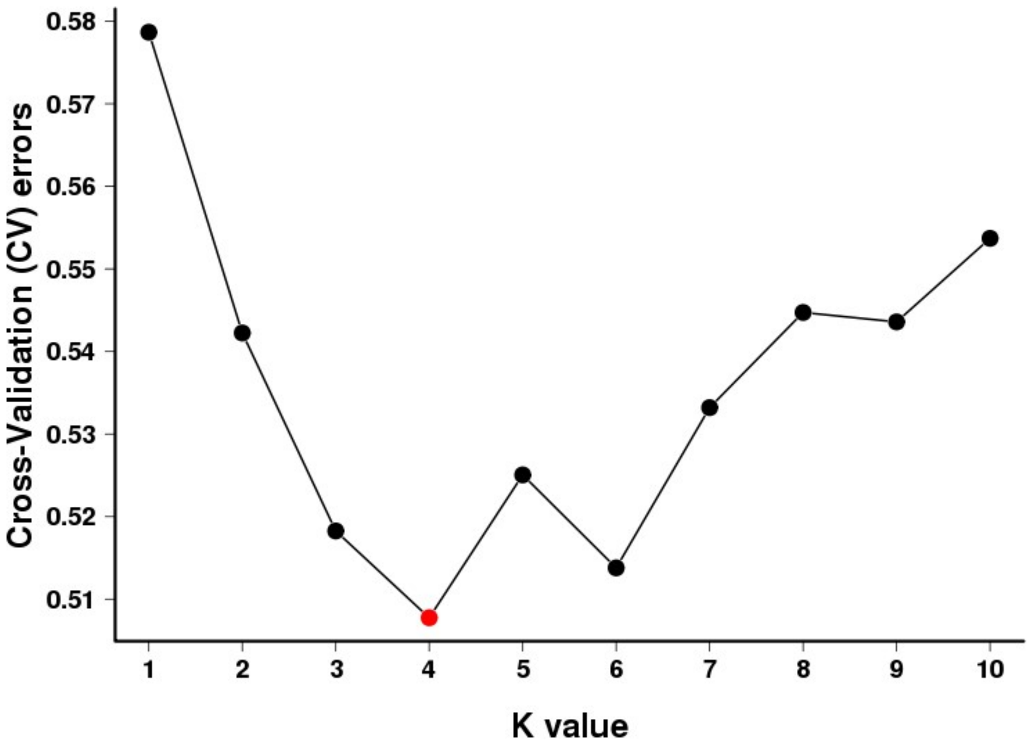

Supplementary Figure 9: Biogeographical ancestry analysis with group value K.

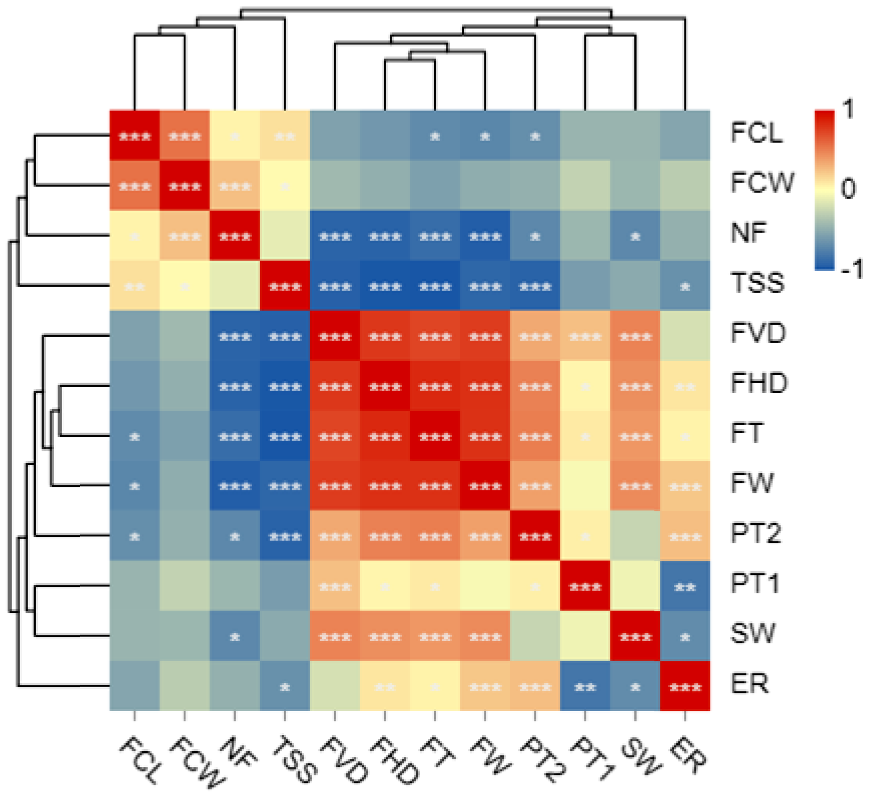

Supplementary Figure 10: Pearson correlation coefficient matrix for analyses of quantitative traits related to fruit quality in 71 germplasms.

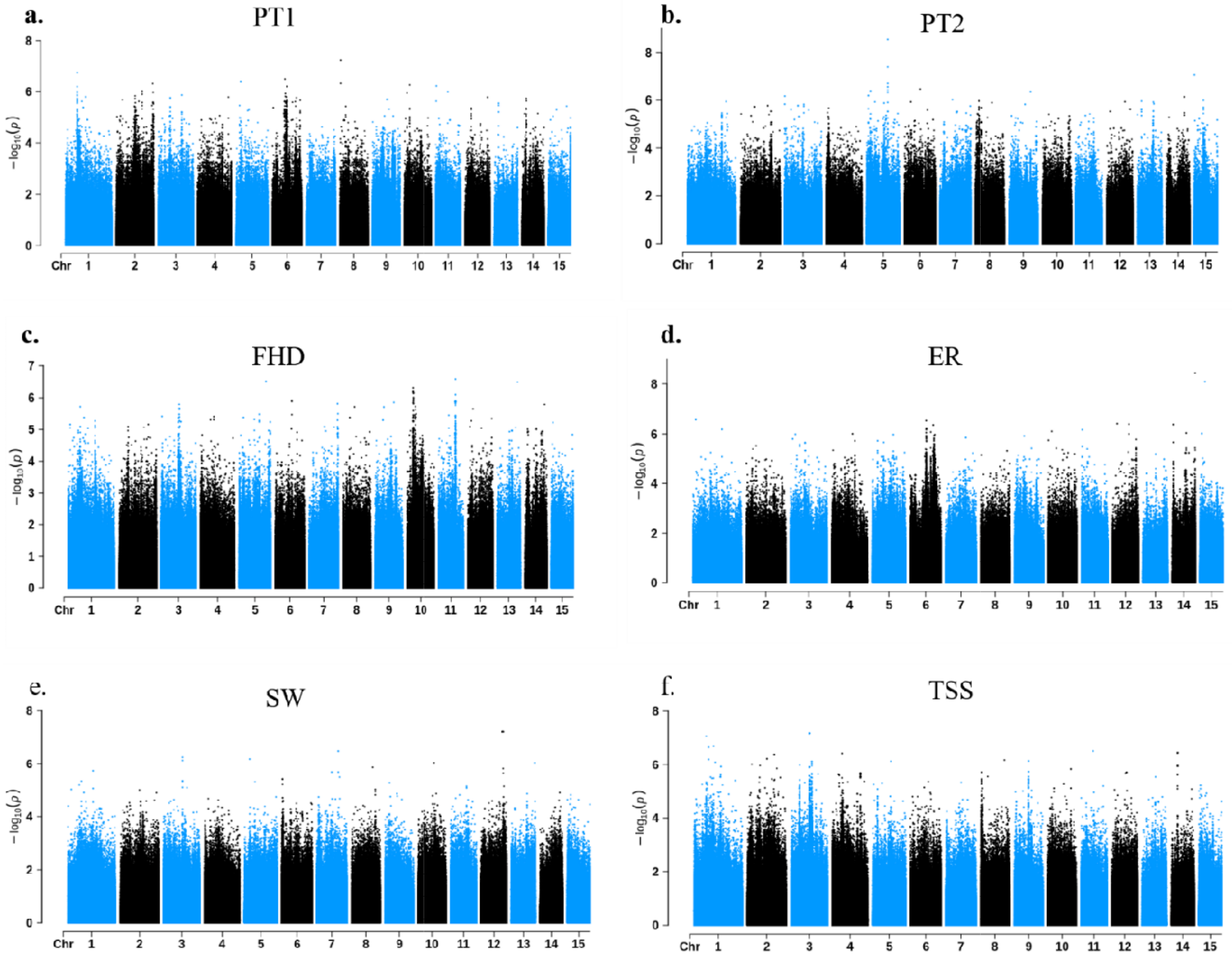

Supplementary Figure 11: Manhattan plot for the genome wide association analysis of longan fruit traits. (a) Fruits' pericarp thickness (PT1). (b) Fruits' pulp thickness (PT2). (c) Fruits' horizontal diameter (FHD). (d) Fruits' edible percentage (ER). (e) Seed weight (SW). (f) Total soluble solid (TSS).

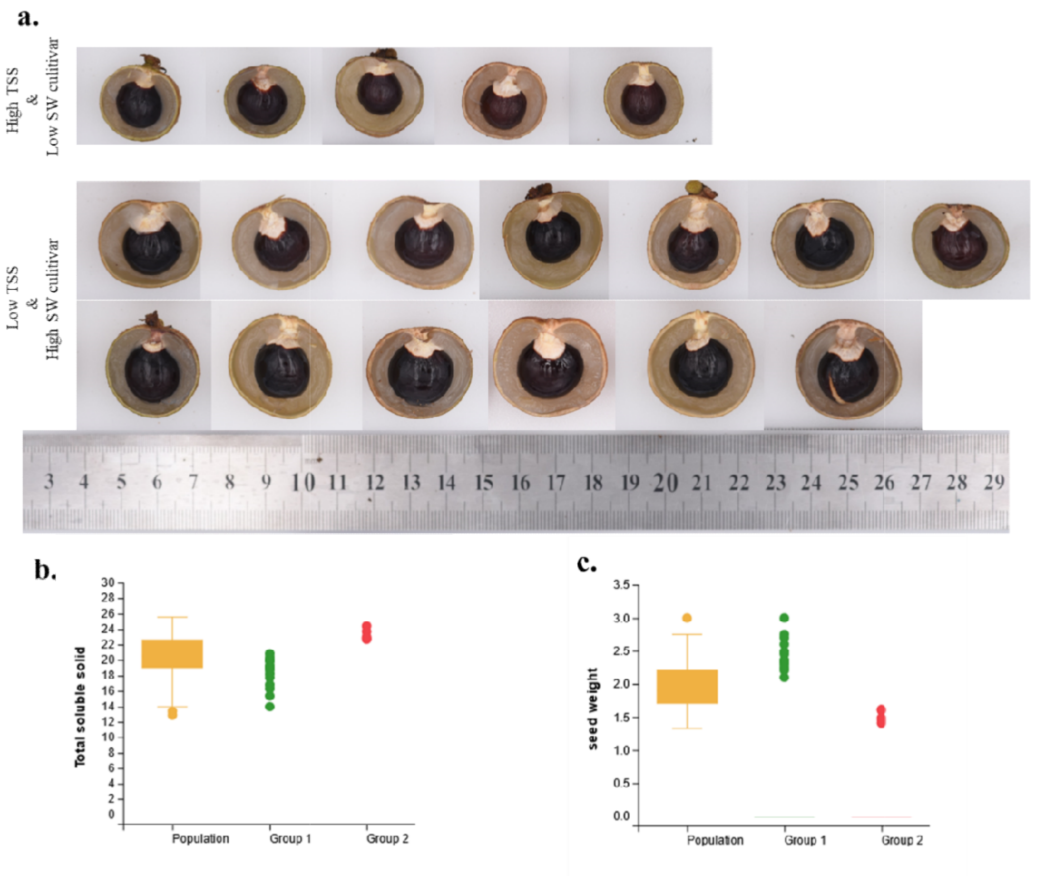

Supplemental Figure 12: Phenotypes of seed and TSS. (a) top: varieties with lower TSS and higher SW, bottom: varieties with higher TSS and lower SW; (b) distribution of TSS among cultivars with photos here; (c) distribution of seed weight among cultivars with photos here.
