# Supplementary material for: Genomic insights into longan evolution from a chromosome-level genome assembly and population genomics of longan accessions
Source: Hortic Res. 2022 Feb 19;9:uhac021. doi: 10.1093/hr/uhac021 (PMC9071379; doi:10.1093/hr/uhac021)
Supplement: Web_Material_uhac021 [file web_material_uhac021.zip › Supplementary Table 1-11.pdf]

Table S1. Sequencing statistics.

#Illumina NGS sequence		
**total base**	**readlen**	**coverage**
25.3G	150bp	56X
**#PacBio**		
**total base**	**Sequences No.**	**Sequences Max.**
184.4G	8452962	441148
	Mean length	N50
	21819	32005
**#HiC**		
**total base**	**readlen**	**coverage**
57.6G	150bp	127X

Table S2. Summary of Illumina data for genome survey and genome polishing.

**Sequences NO.:**	250
**Sequences Min.:**	1,406
**Sequences Max.:**	31,067,541
**Mean length:**	1,821,787
**N50:**	12,096,926
**Total number of bases:**	455,446,992
**GC%**	34%
**Pacbio Reads mapping rate**	89.92%
**Illumina Reads mapping rate**	95.50%
**Genome coverage rate**	94.60%
**BUSCO[genome mode]**	C:96.5%[S:92.6%,D:3.9%],F:1.6%,M:1.9%,n:2121
**LAI index**	20.78
**Complete BUSCOs (C)**	2048
**Complete and single-copy BUSCOs (S)**	1965
**Complete and duplicated BUSCOs (D)**	83
**Fragmented BUSCOs (F)**	33
**Missing BUSCOs (M)**	40
**Total BUSCO groups searched #eudicotyledons_odb10**	2121

Table S3. Gene function annotated by different databases.

	**Annotated number**	**Percent of total genes**
**NR**	35,959	89.00%
**KEGG**	34,152	84.60%
**PlantTFDB**	1,709	4%
**InterPro**	29,661	73.40%

Table S4. Statistics of repetitive elements.

			Number	Length(bp)	% of whole genome
**Class I: Retroelement**	**LTR elements**	**Copia**	54,443	38,850,596	8.552
		**Gypsy**	72,339	70,559,178	15.533
		**other**	10,533	5,561,251	1.224
	**Non-LTR elements**	**LINE**	24,872	8,649,078	1.904
		**SINE**	78	11,196	0.002
		**other**	1,324	367,787	0.081
**Class II:DNA transposon**	**DNA/Helitron**		5,661	2,106,028	0.464
	**DNA/Harbinger**		8,957	3,276,187	0.721
	**DNA/hAT**		36,925	12,647,303	2.784
	**DNA/Crypton**		1,043	241,125	0.053
	**DNA/EnSpm**		23,604	8,370,445	1.843
	**DNA/Mariner**		1,659	401,375	0.088
	**DNA/MuDR**		25,436	11,839,905	2.606
	**DNA/Polinton**		3,214	931,063	0.205
	**DNA/other**		28,163	8,563,583	1.885
**Tandem_Repeat**			1,838	2,025,952	0.446
**Unclassified elements**			93,696	21,041,193	4.632
				189,423,498	41.699

Table S5. Comparison of genes in orthogroups between Dimocarpus longan and 13 other species.

	Amborella trichopod	Ananas_comosus	Arabidopsis_thaliana	Carica_Papaya	Citrus_sinensis	Dimocarpus_longan	Glycine_max	Nicotiana_attenuata	Oryza_sativa	Populus_trichocarpa	Ricinus_communis	Solanum_tuberosum	Theobroma_cacao	Vitis_vinifera
**Number of genes**	27313	27024	27628	27751	25379	40420	55897	33320	35775	41335	31221	39021	21330	29927
**Number of genes in orthogroups**	22563	24366	25219	23072	23709	37401	49595	31860	27899	36440	23922	36101	20984	25725
**Number of unassigned genes**	4750	2658	2409	4679	1670	3019	6302	1460	7876	4895	7299	2920	346	4202
**Percentage of genes in orthogroups**	82.6	90.2	91.3	83.1	93.4	92.5	88.7	95.6	78	88.2	76.6	92.5	98.4	86
**Percentage of unassigned genes**	17.4	9.8	8.7	16.9	6.6	7.5	11.3	4.4	22	11.8	23.4	7.5	1.6	14
**Number of orthogroups**	13258	13108	12871	13480	13498	14712	15148	14262	13871	14466	14349	13979	13063	14107
**Percentage of orthogroups containing species**	45.2	44.6	43.8	45.9	46	50.1	51.6	48.6	47.2	49.3	48.9	47.6	44.5	48
**Number of species-specific orthogroups**	902	711	694	501	227	957	1588	616	1623	702	767	803	103	617
**Number of genes in species-specific orthogroups**	4485	4067	3252	2009	703	6510	6105	3481	6079	2757	2529	8138	435	2046
**Percentage of genes in species-specific orthogroups**	16.4	15	11.8	7.2	2.8	16.1	10.9	10.4	17	6.7	8.1	20.9	2	6.8

Number of species	Number of genes	Number of genes in orthogroups	Number of unassigned genes	Percentage of genes in orthogroups	Percentage of unassigned genes	Number of orthogroups	Number of species-specific orthogroups	Number of genes in species-specific orthogroups	Percentage of genes in species-specific orthogroups	Mean orthogroup size	Median orthogroup size	G50 (assigned genes)	G50 (all genes)	O50 (assigned genes)	O50 (all genes)	Number of orthogroups with all species present	Number of single-copy orthogroups
14	463341	408856	54485	88.2	11.8	29360	10811	52596	11.4	13.9	9	22	20	4901	6199	7530	137

Table S6. List of expanded phenylpropanoid biosynthesis genes and their expression level in different tissues.

#ID	SEQ_Length	Database	Function_ID	Function_Name	root-average expression	leaf-average expression	stem-average expression	fruit-average expression
**D.long005483**	306	Pfam	PF08240	Alcohol dehydrogenase GroES-like domain	0	0	0	0
**D.long024265**	356	Pfam	PF08240	Alcohol dehydrogenase GroES-like domain	0	0.06	0	0.026666667
**D.long030408**	355	Pfam	PF08240	Alcohol dehydrogenase GroES-like domain	282.4866667	0.153333333	43.41666667	16.1
**D.long030412**	287	Pfam	PF08240	Alcohol dehydrogenase GroES-like domain	32.70666667	1.476666667	77.27333333	26.30333333
**D.long030414**	354	Pfam	PF08240	Alcohol dehydrogenase GroES-like domain	32.08333333	113.3433333	18.26	51.70333333
**D.long030415**	355	Pfam	PF08240	Alcohol dehydrogenase GroES-like domain	203.8633333	3.86	33.23333333	22.76
**D.long031881**	362	Pfam	PF08240	Alcohol dehydrogenase GroES-like domain	201.3966667	1.946666667	207.4433333	45.2
**D.long031882**	360	Pfam	PF08240	Alcohol dehydrogenase GroES-like domain	0.653333333	0	0.116666667	6.78
**D.long031883**	362	Pfam	PF08240	Alcohol dehydrogenase GroES-like domain	1.03	3.546666667	9.786666667	2.1
**D.long031884**	361	Pfam	PF08240	Alcohol dehydrogenase GroES-like domain	1.543333333	0.21	1.436666667	3.733333333
**D.long031885**	346	Pfam	PF08240	Alcohol dehydrogenase GroES-like domain	2.85	0	1.143333333	0.706666667
**D.long031886**	362	Pfam	PF08240	Alcohol dehydrogenase GroES-like domain	19.00333333	0.243333333	3.29	7.24
**D.long031887**	362	Pfam	PF08240	Alcohol dehydrogenase GroES-like domain	1.523333333	0.806666667	22.91666667	3.57
**D.long031892**	361	Pfam	PF08240	Alcohol dehydrogenase GroES-like domain	86.49333333	0	4.226666667	0.153333333
**D.long032679**	360	Pfam	PF08240	Alcohol dehydrogenase GroES-like domain	0	154.7566667	1.953333333	81.24
**D.long032684**	359	Pfam	PF08240	Alcohol dehydrogenase GroES-like domain	5.373333333	5.913333333	16.55	16.65666667
**D.long003186**	501	Pfam	PF00171	Aldehyde dehydrogenase family	0.156666667	2.696666667	0.873333333	0.303333333
**D.long014098**	501	Pfam	PF00171	Aldehyde dehydrogenase family	1.046666667	105.1966667	19.39	146.3666667
**D.long002765**	548	Pfam	PF00501	AMP-binding enzyme	96.25	55.25333333	86.36	36.34333333
**D.long011121**	540	Pfam	PF00501	AMP-binding enzyme	6.646666667	159.22	119.8133333	63.55333333
**D.long011346**	540	Pfam	PF00501	AMP-binding enzyme	211.4266667	191.85	139.7733333	78.69333333
**D.long014725**	580	Pfam	PF00501	AMP-binding enzyme	13.83	29.06	74.93	76.85333333
**D.long024147**	493	TIGRFAM	TIGR03356	BGL: beta-galactosidase	74.56	90.94333333	128.1166667	125
**D.long024148**	493	TIGRFAM	TIGR03356	BGL: beta-galactosidase	100.47	18.82333333	19.98333333	56.21666667
**D.long024149**	493	TIGRFAM	TIGR03356	BGL: beta-galactosidase	183.3133333	7.606666667	55.73	64.91666667
**D.long019626**	557	Pfam	PF00232	Glycosyl hydrolase family 1	6.49	8.703333333	10.32666667	9.886666667
**D.long021797**	120	Pfam	PF00232	Glycosyl hydrolase family 1	0	0	0	0
**D.long021814**	502	Pfam	PF00232	Glycosyl hydrolase family 1	0	0	0	0
**D.long021816**	511	Pfam	PF00232	Glycosyl hydrolase family 1	0.023333333	0	0	0
**D.long021818**	514	Pfam	PF00232	Glycosyl hydrolase family 1	0.813333333	0.563333333	0.046666667	12.5
**D.long025080**	514	Pfam	PF00232	Glycosyl hydrolase family 1	0.383333333	0	3.606666667	0
**D.long025081**	828	Pfam	PF00232	Glycosyl hydrolase family 1	11.63666667	0.59	30.6	2.04
**D.long025824**	315	Pfam	PF00232	Glycosyl hydrolase family 1	0	0.076666667	0	0
**D.long025826**	524	Pfam	PF00232	Glycosyl hydrolase family 1	1.866666667	0.036666667	0.466666667	0.453333333
**D.long026156**	524	Pfam	PF00232	Glycosyl hydrolase family 1	1.903333333	0	0.243333333	0.673333333
**D.long026162**	222	Pfam	PF00232	Glycosyl hydrolase family 1	0	0	0	0
**D.long028671**	618	Pfam	PF00232	Glycosyl hydrolase family 1	6.723333333	12.68	9.346666667	5.65
**D.long028672**	525	Pfam	PF00232	Glycosyl hydrolase family 1	0.576666667	49.13	7.92	3.413333333
**D.long028674**	535	Pfam	PF00232	Glycosyl hydrolase family 1	4.813333333	60.19	5.996666667	25.4
**D.long028675**	510	Pfam	PF00232	Glycosyl hydrolase family 1	0	5.316666667	1.533333333	14.73333333
**D.long035562**	161	Pfam	PF00232	Glycosyl hydrolase family 1	0.256666667	0	0	0.1
**D.long035566**	520	Pfam	PF00232	Glycosyl hydrolase family 1	6	40.46	5.683333333	14.08
**D.long037807**	500	Pfam	PF00232	Glycosyl hydrolase family 1	0.11	0.036666667	0.056666667	0.053333333
**D.long037808**	500	Pfam	PF00232	Glycosyl hydrolase family 1	0.773333333	0.043333333	0.766666667	0.406666667
**D.long037809**	494	Pfam	PF00232	Glycosyl hydrolase family 1	10.44	0	0.433333333	0.023333333
**D.long001393**	513	PRINTS	PR00131	Glycosyl hydrolase family 1 signature	0.116666667	0	0	1.933333333
**D.long009526**	521	PRINTS	PR00131	Glycosyl hydrolase family 1 signature	1.833333333	47.78666667	22.01333333	25.31333333
**D.long010988**	515	PRINTS	PR00131	Glycosyl hydrolase family 1 signature	16.18666667	0	3.546666667	11.00333333
**D.long011003**	513	PRINTS	PR00131	Glycosyl hydrolase family 1 signature	13.69666667	0.016666667	0.366666667	2.113333333
**D.long019625**	527	PRINTS	PR00131	Glycosyl hydrolase family 1 signature	8.603333333	1.02	5.043333333	7.313333333
**D.long032044**	515	PRINTS	PR00131	Glycosyl hydrolase family 1 signature	28.65333333	0	0.086666667	0.996666667
**D.long004123**	238	Pfam	PF01596	O-methyltransferase	38.20333333	11.58333333	21.68333333	29.74666667
**D.long004124**	238	Pfam	PF01596	O-methyltransferase	43.85	18.47666667	28.08	34.45
**D.long004126**	238	Pfam	PF01596	O-methyltransferase	138.8866667	11.40333333	79.62666667	28.78666667
**D.long000403**	320	Pfam	PF00141	Peroxidase	76.15666667	0.073333333	0.24	0.036666667
**D.long000404**	335	Pfam	PF00141	Peroxidase	0	0	0	0
**D.long000406**	373	Pfam	PF00141	Peroxidase	0	0	0	0
**D.long004529**	322	Pfam	PF00141	Peroxidase	32.2	18.20666667	25.33666667	45.27333333
**D.long006178**	336	Pfam	PF00141	Peroxidase	0	0	0	0
**D.long006180**	338	Pfam	PF00141	Peroxidase	6.576666667	0.123333333	1.11	1.46
**D.long006181**	337	Pfam	PF00141	Peroxidase	2.996666667	0	2.51	2.136666667
**D.long006182**	130	Pfam	PF00141	Peroxidase	0	0	0	0
**D.long006183**	338	Pfam	PF00141	Peroxidase	0.086666667	0	3.403333333	0.576666667
**D.long006184**	338	Pfam	PF00141	Peroxidase	0.086666667	0	2.623333333	0.883333333
**D.long006185**	335	Pfam	PF00141	Peroxidase	0	0.073333333	0.193333333	0.133333333
**D.long006479**	322	Pfam	PF00141	Peroxidase	1.18	0.383333333	1.2	0
**D.long012043**	326	Pfam	PF00141	Peroxidase	0	1.466666667	0.21	0.113333333
**D.long012051**	324	Pfam	PF00141	Peroxidase	0.05	762.3133333	0.32	0
**D.long012053**	336	Pfam	PF00141	Peroxidase	4.04	486.1566667	61.42	225.7366667
**D.long012056**	332	Pfam	PF00141	Peroxidase	0	0.58	5.89	3.016666667
**D.long012057**	327	Pfam	PF00141	Peroxidase	13.11	1.313333333	12.95333333	3.603333333
**D.long012540**	281	Pfam	PF00141	Peroxidase	0	2.24	0.26	0
**D.long014332**	422	Pfam	PF00141	Peroxidase	0.03	0	0	1.793333333
**D.long014333**	330	Pfam	PF00141	Peroxidase	0	0	0	0
**D.long014334**	322	Pfam	PF00141	Peroxidase	0	0	0	0
**D.long024588**	366	Pfam	PF00141	Peroxidase	0	0	0	0
**D.long025054**	320	Pfam	PF00141	Peroxidase	25.18666667	0	1.766666667	0.88
**D.long025112**	318	Pfam	PF00141	Peroxidase	0.05	0	0	0
**D.long029149**	330	Pfam	PF00141	Peroxidase	0	0	0	0
**D.long029152**	329	Pfam	PF00141	Peroxidase	0.473333333	0	0	0.323333333
**D.long029153**	329	Pfam	PF00141	Peroxidase	0.793333333	8.89	3.423333333	3.433333333
**D.long032015**	275	Pfam	PF00141	Peroxidase	6.296666667	1.806666667	12.55	20.28333333
**D.long032016**	331	Pfam	PF00141	Peroxidase	1.036666667	2.306666667	0.236666667	3.883333333
**D.long034018**	338	Pfam	PF00141	Peroxidase	11.76	1.583333333	1.253333333	2.363333333
**D.long035259**	337	Pfam	PF00141	Peroxidase	0.036666667	0	0	0
**D.long035294**	339	Pfam	PF00141	Peroxidase	0	0	0.03	0.103333333
**D.long037726**	353	Pfam	PF00141	Peroxidase	35.64333333	62.85666667	46.55	55.29333333
**D.long037727**	353	Pfam	PF00141	Peroxidase	63.76666667	188.9866667	68.08666667	135.3533333
**D.long037728**	314	Pfam	PF00141	Peroxidase	0	0	0	0.193333333
**D.long037729**	320	Pfam	PF00141	Peroxidase	0.146666667	0.11	0	1.503333333
**D.long037730**	322	Pfam	PF00141	Peroxidase	4.276666667	2.556666667	0.643333333	1.203333333
**D.long037732**	320	Pfam	PF00141	Peroxidase	0	0	0	0
**D.long005200**	709	TIGRFAM	TIGR01226	phe_am_lyase: phenylalanine ammonia-lyase	269.7766667	403.8066667	291.8966667	80.39666667
**D.long007573**	706	TIGRFAM	TIGR01226	phe_am_lyase: phenylalanine ammonia-lyase	44.87	88.78333333	40.91333333	22.40666667
**D.long007574**	702	TIGRFAM	TIGR01226	phe_am_lyase: phenylalanine ammonia-lyase	24.70666667	1.01	12.5	6.973333333
**D.long007575**	706	TIGRFAM	TIGR01226	phe_am_lyase: phenylalanine ammonia-lyase	1.416666667	0	0.036666667	0.016666667
**D.long034387**	725	TIGRFAM	TIGR01226	phe_am_lyase: phenylalanine ammonia-lyase	135.7666667	139.9366667	383.6133333	293.9133333

Table S7. List of different expressed IPR enriched gene families.

#IPR id	IPR description
IPR002213	UDP-glucuronosyl/UDP-glucosyltransferase
IPR002401	Cytochrome P450, E-class, group I
IPR000719	Protein kinase domain
IPR036396	Cytochrome P450 superfamily
IPR001128	Cytochrome P450
IPR036852	Peptidase S8/S53 domain superfamily
IPR000209	Peptidase S8/S53 domain
IPR002160	Proteinase inhibitor I3, Kunitz legume
IPR000858	S-locus glycoprotein domain
IPR021720	Malectin domain
IPR010259	Peptidase S8 propeptide/proteinase inhibitor I9
IPR000726	Glycoside hydrolase, family 19, catalytic
IPR020843	Polyketide synthase, enoylreductase domain
IPR025287	Wall-associated receptor kinase, galacturonan-binding domain
IPR005123	Oxoglutarate/iron-dependent dioxygenase

Table S8. List of UGTs genes ID and their expression level.

#transcript_id	gene_id	leaf	root	stem	fruit
**D.long000750.01**	D.long000750	14.56	0.18	17.91667	19.68
**D.long000751.01**	D.long000751	3.97	3.035	4.113333	13.285
**D.long000752.01**	D.long000752	0.82	33.32	7.256667	2.27
**D.long000753.01**	D.long000753	24.74667	79.92	29.59667	33.4
**D.long000754.01**	D.long000754	0.066667	30.06	0.49	0.385
**D.long001253.01**	D.long001253	12.57	0.04	0.803333	0.03
**D.long001256.01**	D.long001256	14.22333	7.055	1.383333	3.585
**D.long002736.01**	D.long002736	5.796667	4.31	7.143333	12.69
**D.long004697.01**	D.long004697	0.023333	0.04	0.506667	0.395
**D.long005887.01**	D.long005887	13.21	18.305	21.78333	35.795
**D.long005889.01**	D.long005889	7.253333	20.47	11.41667	20.53
**D.long005890.01**	D.long005890	0.623333	14.395	3.55	5.945
**D.long005892.01**	D.long005892	369.6867	313.02	183.1533	182.915
**D.long007001.01**	D.long007001	0.26	3.44	0.773333	0
**D.long007002.01**	D.long007002	15.17333	0.23	17.84	8.14
**D.long007337.01**	D.long007337	0	0	0	0.12
**D.long007914.01**	D.long007914	0.63	0	0.02	13.995
**D.long007915.01**	D.long007915	0	0.04	0	1.285
**D.long007934.01**	D.long007934	15.66	51.695	48.11667	37.99
**D.long007936.01**	D.long007936	0.346667	4.42	5.473333	2.555
**D.long007938.01**	D.long007938	5.53	3.545	0.753333	0.55
**D.long007941.01**	D.long007941	0.303333	0.545	1.233333	1.38
**D.long007942.01**	D.long007942	3.093333	0.855	13.17667	9.065
**D.long007958.01**	D.long007958	0.226667	0	0	0.08
**D.long007959.01**	D.long007959	0.066667	0.36	0.02	0
**D.long008122.01**	D.long008122	0.046667	24.275	2.89	3.9
**D.long008123.01**	D.long008123	0.02	5.495	1.283333	0.835
**D.long008126.01**	D.long008126	0	0.95	0	0
**D.long008130.01**	D.long008130	1.646667	0.505	0.46	1.635
**D.long008131.01**	D.long008131	12.77	10.26	6.163333	13.235
**D.long008459.01**	D.long008459	2.873333	23.325	5.913333	1.62
**D.long009885.01**	D.long009885	0	0	0.053333	0
**D.long011239.01**	D.long011239	56.57667	0.885	97.63	49.335
**D.long013435.01**	D.long013435	0.316667	46.305	3.84	5.25
**D.long015203.01**	D.long015203	0.243333	0	0	0.12
**D.long015746.01**	D.long015746	8.88	7.09	1.14	2.2
**D.long016471.01**	D.long016471	7.073333	8.7	10.35667	14.375
**D.long016946.01**	D.long016946	0.406667	0.1	0.263333	0.085
**D.long017794.01**	D.long017794	0.643333	0.93	0.303333	0.215
**D.long019494.01**	D.long019494	3.79	24.95	68.02333	40.25
**D.long019495.01**	D.long019495	11.6	0.22	3.193333	3.165
**D.long019496.01**	D.long019496	0	27.97	0.063333	0.055
**D.long019506.01**	D.long019506	0	2.405	1.1	0.25
**D.long019508.01**	D.long019508	0.146667	6.765	1.443333	0.93
**D.long019513.01**	D.long019513	0.11	16.505	0	0.135
**D.long020005.01**	D.long020005	0	0.05	0	0
**D.long020006.01**	D.long020006	0	4.515	0.883333	0.265
**D.long020009.01**	D.long020009	0	1.45	0.823333	0.725
**D.long022372.01**	D.long022372	0.016667	0	0.443333	0.85
**D.long023017.01**	D.long023017	0.18	0.04	0.083333	0.025

**D.long023291.01**	D.long023291	1.1	16.33	5.62	6.15
**D.long023303.01**	D.long023303	31.83	30.09	58.61	70.03
**D.long023305.01**	D.long023305	0.07	0	0.443333	2.63
**D.long023307.01**	D.long023307	0	0	0.02	0.285
**D.long023309.01**	D.long023309	0.523333	21.8	2.81	63.75
**D.long024681.01**	D.long024681	3.493333	0.07	0.17	0
**D.long026389.01**	D.long026389	1.073333	12.585	5.703333	4.31
**D.long027024.01**	D.long027024	0.436667	4.46	5.92	6.515
**D.long027795.01**	D.long027795	0	0	0.226667	0
**D.long027797.01**	D.long027797	0.763333	19.455	1.323333	1.8
**D.long027798.01**	D.long027798	22.66667	8.01	16.66	12.64
**D.long030886.01**	D.long030886	23.56333	79.455	78.65	60.97
**D.long030887.01**	D.long030887	5.773333	30.575	58.18667	34.54
**D.long033649.01**	D.long033649	0.553333	3.045	1.306667	8.08
**D.long033685.01**	D.long033685	0.033333	5.245	0	3.73
**D.long033689.01**	D.long033689	0.793333	10.27	1.626667	0.175
**D.long033693.01**	D.long033693	0.296667	2.835	0.156667	4.745
**D.long033697.01**	D.long033697	0.14	1.975	0.193333	0.86
**D.long033704.01**	D.long033704	6.91	25.825	10.51667	0.4
**D.long033705.01**	D.long033705	5.44	1.66	21.44333	33.305
**D.long033707.01**	D.long033707	1.573333	3.825	14.85	47.005
**D.long033718.01**	D.long033718	0.583333	1.095	7.45	8.475
**D.long033719.01**	D.long033719	146.17	0	11.24333	1.48
**D.long033721.01**	D.long033721	1.393333	0.115	0	0.025
**D.long034281.01**	D.long034281	0.14	1.835	0.603333	0.345
**D.long034282.01**	D.long034282	0	0.035	0	0.075
**D.long034283.01**	D.long034283	0.29	31	3.61	13.985
**D.long034284.01**	D.long034284	9.436667	12.545	9.266667	13.795
**D.long034285.01**	D.long034285	0	0	0.056667	0.355
**D.long034286.01**	D.long034286	3.4	0	3.013333	3.575
**D.long034287.01**	D.long034287	0.06	0	0.053333	0
**D.long034289.01**	D.long034289	1.396667	0.855	13.27	10.35
**D.long034290.01**	D.long034290	0.64	4.975	6.203333	2.355
**D.long034371.01**	D.long034371	0.02	1.06	0	0.335
**D.long036345.01**	D.long036345	0.146667	0.19	0.07	12.725
**D.long036346.01**	D.long036346	23.62333	65.915	51.52333	42.67
**D.long036348.01**	D.long036348	0.023333	0.695	0.06	29.235
**D.long036350.01**	D.long036350	0.64	0.39	0.15	4.395
**D.long036353.01**	D.long036353	0.316667	6.125	0.69	20.27
**D.long036354.01**	D.long036354	0.066667	0.495	0.233333	7.195
**D.long036357.01**	D.long036357	1.173333	1.8	1.616667	2.845
**D.long036906.01**	D.long036906	0	0.96	0	4.9
**D.long036908.01**	D.long036908	0.423333	1.46	0.596667	14.105
**D.long036910.01**	D.long036910	0.653333	7	3.053333	8.095
**D.long037404.01**	D.long037404	0	0	0	0.135
**D.long037439.01**	D.long037439	1.433333	2.83	2.046667	0.175

Table S9. List of genome resequencing samples and their locations.

Sequencing-ID	First letter of cultivar and its location	Location (Province/Country)	City	Artificial Breeding
D01	CL-GD	GuangDong	GaoZhou	No
D02	SMM-GD	GuangDong	GaoZhou	No
D03	BHM-GD	GuangDong	GaoZhou	No
D04	LSM-GD	GuangDong	GaoZhou	No
D05	TBM-GD	GuangDong	GaoZhou	No
D06	SLM-GD	GuangDong	GaoZhou	No
D07	HH-GD	GuangDong	GaoZhou	No
D08	GSEH-GD	GuangDong	JieYang	No
D09	WGTGY-GD	GuangDong	GaoZhou	No
D10	JRY-GD	GuangDong	GaoZhou	No
D11	HLGY-GD	GuangDong	GaoZhou	No
D12	JSY-GD	GuangDong	GaoZhou	No
D13	SLR-GD	GuangDong	GaoZhou	No
D14	HDGY-GD	GuangDong	GaoZhou	No
D15	YC-GD	GuangDong	GuangZhou	Yes
D16	GZ-GD	GuangDong	GuangZhou	Yes
D17	ZBL-GX	GuangXi	GuiPing	No
D18	SSCR-GD	GuangDong	GuangZhou	Yes
D19	CHDGY-GD	GuangDong	CongHua	No
D20	XJWY-GD	GuangDong	GuangZhou	Yes
D21	LZZ-GD	GuangDong	JieYang	No
D22	SSCS-GD	GuangDong	GuangZhou	Yes
D23	XJYH-GD	GuangDong	GuangZhou	Yes
D24	SX-GD	GuangDong	NanHai	No
D25	SY-GD	GuangDong	ZhongShan	No
D26	DWY-GX	GuangXi	YuLin	No
D27	JY-GD	GuangDong	GuangZhou	Yes
D28	KHL-Aus	Australia	unknown	No
D29	YD-Thai	Thailand	unknown	No
D30	PT-Thai	Thailand	unknown	No
D31	XY-GD	GuangDong	GuangZhou	Yes
D32	TXZQ-GX	GuangXi	WuZhou	No
D33	YZDWY-GX	GuangXi	YuLin	No
D34	GZA1-GD	GuangDong	GuangZhou	Yes
D35	GZ3-GD	GuangDong	GuangZhou	Yes
D36	DWYH-GD	GuangDong	DongGuan	No
D37	DF-GD	GuangDong	DongGuan	No
D38	LY-GD	GuangDong	GuangZhou	Yes
D39	TGSJ-Thai	Thailand	unknown	No
D40	YTB-FJ	FuJian	PuTian	No
D41	SZP-Thai	Thailand	unknown	No
D42	JL-FJ	FuJian	PuTian	No
D43	HXB-FJ	FuJian	PuTian	No
D44	QKBY-FJ	FuJian	ChangLe	No
D45	SZ-FJ	FuJian	XiaMen	No
D46	WCL-FJ	FuJian	ChangLe	No
D47	PMA-FJ	FuJian	PuTian	No
D48	GX-GX	GuangXi	NanNing	Yes
D49	SG-SC	SiChuan	LuZhou	No
D50	JYW-FJ	FuJian	PuTian	No
D51	LY-FJ	FuJian	PuTian	No
D52	LDB-FJ	FuJian	PuTian	No
D54	GLZ-GX	GuangXi	NanNing	No
D55	LF-SC	SiChuan	LuZhou	No
D56	CHZ-GD	GuangDong	JieYang	No
D57	CPZ-GD	GuangDong	RaoPing	No
D58	CK-FJ	FuJian	XiaMen	No
D59	GMB-FJ	FuJian	PuTian	No
D60	SNYH-FJ	FuJian	PuTian	No
D61	HBP-FJ	FuJian	TongAn	No
D62	HKZ-FJ	FuJian	unknown	No
D63	HY-GD	GuangDong	Taishan	No
D64	CSB-FJ	FuJian	PuTian	No
D65	QZB-FJ	FuJian	PuTian	No
D66	WLL-FJ	FuJian	XianYou	No
D67	JDB-FJ	FuJian	PuTian	No
D68	ZTB-FJ	FuJian	QuanZhou	No
D69	LQB-FJ	FuJian	PuTian	No
D70	FLD	GuangDong	ChaoZhou	No
D71	BYZ-FJ	FuJian	TongAn	No
D72	YY106-FJ	FuJian	PuTian	No
D73	QKJ-FJ	FuJian	unknown	No
D74	SFB-FJ	FuJian	PuTian	No
D75	GHW	GuangDong	GuangZhou	Yes
D76	FY-FJ	FuJian	FuZhou	No
D77	JH-GD	GuangDong	GuangZhou	Yes
D78	LZ-SC	SiChuan	LuZhou	No
D79	GZA2-GD	GuangDong	GuangZhou	Yes
D80	YNSJ-Viet	Vietnam	unknown	No
D81	FLS-FJ	FuJian	XiaMen	No
D82	FLHK-SC	SiChuan	ChongQing	No
D83	FDWM-GD	GuangDong	ChaoZhou	Yes
D84	LY-HN	HaiNan	unknown	No
D85	GZ2-GD	GuangDong	GuangZhou	Yes
D86	DB-FJ	FuJian	QuanZhou	No
D87	GMYH-GX	GuangXi	NanNing	Yes
D88	HXZ-FJ	FuJian	ChangLe	No

Table S10. GWAS results of significant SNPs associated with six traits and gene annotations .

Trait	Chromosome	Gene id	Promoter	Intron	CDS	Synonymous	Nonsynonymous	Swiss.port.anno
**Pericarp Thickness**	chr1	D.long001288.01	7	0	0	0	0	sp|F4JKK0|SUD1_ARATH
		D.long001288.02	0	46	6	3	3	sp|F4JKK0|SUD1_ARATH
		D.long001289.01	13	141	20	14	6	unknown
		D.long001290.01	12	18	4	2	2	unknown
		D.long001291.01	1	23	0	0	0	unknown
		D.long001292.01	2	106	26	10	16	unknown
		D.long001293.01	27	0	266	58	208	sp|Q9T048|DRL27_ARATH
		D.long001294.01	70	13	109	34	75	sp|Q8L3R3|RFL1_ARATH
		D.long001295.01	10	0	69	22	47	unknown
		D.long001296.01	1	108	273	273	0	unknown
		D.long001297.01	11	0	41	8	33	sp|O81825|DRL28_ARATH
		D.long001298.01	33	10	55	16	39	sp|O81825|DRL28_ARATH
		D.long001299.01	10	240	34	11	23	unknown
		D.long001300.01	2	11	35	8	27	unknown
		D.long001301.01	16	75	6	2	4	unknown
		D.long001302.01	4	54	8	2	6	unknown
		D.long001303.01	3	68	7	2	5	unknown
		D.long001304.01	5	0	14	6	8	sp|Q9LNE6|U89C1_ARATH
		D.long001305.01	23	0	17	8	9	sp|Q9LNE6|U89C1_ARATH
		D.long001306.01	20	110	5	1	4	unknown
		D.long001307.01	30	9	31	11	20	unknown
		D.long001308.01	1	19	12	3	9	sp|Q9LRQ8|PMAT2_ARATH
		D.long001309.01	5	2	1	1	0	sp|O04421|SRP14_ARATH
		D.long001310.01	25	15	5	2	3	unknown
		D.long001311.01	14	60	15	8	7	unknown
	chr6	D.long025467.01	39	2	23	10	13	unknown
		D.long025468.01	11	0	27	11	16	sp|Q9M219|MTEFH_ARATH
		D.long025469.01	38	32	85	36	49	unknown
		D.long025470.01	4	18	5	2	3	sp|Q9SY60|EX84C_ARATH
		D.long025471.01	0	144	44	14	30	unknown
		D.long025472.01	19	0	13	4	9	sp|Q9FJK3|AGL80_ARATH
		D.long025473.01	1	30	51	15	36	unknown
		D.long025474.01	60	11	68	14	54	unknown
		D.long025475.01	18	56	11	10	1	sp|Q9FVV1|GDL28_ARATH
		D.long025476.01	5	4	23	6	17	sp|Q9XHM1|EIF3C_MEDTR
		D.long025477.01	3	66	28	11	17	sp|Q9FKE9|RHD32_ARATH

Trait	Chromosome	Gene id	Promoter	Intron	CDS	Synonymous	Nonsynonymous	Swiss.port.anno
**Pulp Thickness**	chr5	D.long015104.01	17	4	14	5	9	unknown
		D.long015105.01	15	1	84	29	55	sp|C0LGP4|Y3475_ARATH
		D.long015106.01	40	6	42	30	12	unknown
		D.long015107.01	0	0	56	56	0	unknown
		D.long015108.01	31	11	36	14	22	unknown
		D.long015109.01	5	29	42	10	32	unknown
		D.long015110.01	10	70	44	9	35	sp|C0LGT6|EFR_ARATH
		D.long015111.01	6	5	32	8	24	sp|Q8RWZ5|SD25_ARATH
		D.long015112.01	28	13	30	8	22	sp|Q39688|EP1G_DAUCA
		D.long015113.01	29	0	42	10	32	sp|O82777|SBT3_SOLLC
		D.long015114.01	0	0	29	18	11	sp|O82777|SBT3_SOLLC
		D.long015115.01	27	0	17	2	15	unknown
		D.long015116.01	5	0	4	1	3	sp|O82777|SBT3_SOLLC
		D.long015117.01	24	7	20	5	15	unknown
		D.long015118.01	20	7	17	10	7	sp|A0A1D6F9Y9|C92C6_MAIZE

Trait	Chromosome	Gene id	Promoter	Intron	CDS	Synonymous	Nonsynonymous	Swiss.port.anno
**Fruit' Horizontal Diameter**	chr10	D.long035939.01	3	18	6	5	1	sp|Q43594|TBB1_ORYSJ
		D.long035940.01	1	32	7	7	0	sp|F4IIZ9|SCAB2_ARATH
		D.long035941.01	4	0	18	4	14	sp|Q4PSU4|AGL61_ARATH
		D.long035942.01	14	8	21	6	15	sp|P25011|CCNB1_SOYBN
		D.long035943.01	17	57	54	14	40	sp|F4JT80|RPP2B_ARATH
		D.long035944.01	1	14	0	0	0	sp|Q6R8G7|PHO13_ARATH
		D.long035945.01	6	1	5	0	5	sp|Q9LET7|CI111_ARATH
		D.long035946.01	14	0	5	3	2	sp|O22210|MYBC1_ARATH
		D.long035947.01	1	81	7	3	4	unknown
		D.long035948.01	14	0	7	3	4	sp|O64703|AGL29_ARATH
		D.long035949.01	5	8	12	3	9	unknown
		D.long035950.02	14	1	7	3	4	unknown
	chr11	D.long027632.01	15	7	6	4	2	unknown
		D.long027633.01	2	5	6	2	4	unknown
		D.long027634.01	2	0	1	0	1	unknown
		D.long027635.01	5	1	3	2	1	unknown
		D.long027636.01	9	1	6	1	5	unknown
		D.long027637.01	0	0	1	0	1	sp|Q9C9A2|PP112_ARATH
		D.long027638.01	0	0	1	0	1	sp|Q9FGW0|FLA20_ARATH
		D.long027639.01	5	3	0	0	0	sp|Q8VYP9|ICML1_ARATH
		D.long027640.01	0	2	2	0	2	sp|Q93ZF5|PHO11_ARATH
		D.long027641.01	3	3	0	0	0	unknown
		D.long027642.01	0	0	1	1	0	unknown
		D.long027643.01	9	2	12	5	7	sp|Q9S7I0|TADA_ARATH
		D.long027644.01	44	2	38	7	31	unknown
		D.long027645.01	0	0	2	0	2	unknown
		D.long027646.01	65	89	97	97	0	unknown
		D.long027647.01	11	39	16	11	5	sp|Q9SX33|ALA9_ARATH
		D.long027648.01	0	1	2	1	1	unknown
		D.long027649.01	12	0	2	2	0	sp|Q9LI84|CML16_ARATH
		D.long027650.01	3	0	6	4	2	unknown
		D.long027651.01	3	9	10	4	6	sp|Q9C660|PEK10_ARATH
		D.long027652.01	4	49	9	2	7	unknown
		D.long027653.01	14	0	14	5	9	sp|Q9SY20|FB20_ARATH
		D.long027654.01	11	0	14	4	10	sp|Q9SSR4|PPR77_ARATH
		D.long027655.01	7	0	4	0	4	sp|Q9SI09|XERIC_ARATH
		D.long027656.01	4	2	11	5	6	sp|Q9ZT94|POLR2_ARATH
		D.long027657.01	5	0	7	1	6	sp|Q9SI09|XERIC_ARATH

Trait	Chromosome	Gene id	Promoter	Intron	CDS	Synonymous	Nonsynonymous	Swiss.port.anno
**Edible Rate**	chr14	D.long030308.01	0	4	8	5	3	sp|Q9SJG9|MPK20_ARATH
		D.long030309.01	37	137	55	20	35	sp|P0C2F6|RNHX1_ARATH
		D.long030310.01	3	0	7	4	3	sp|Q9SJH0|PAR1_ARATH
		D.long030311.01	24	0	13	6	7	sp|Q9LXR8|PMD1_ARATH
		D.long030312.01	12	3	26	9	17	unknown
		D.long030313.01	30	0	37	17	20	unknown
		D.long030314.01	44	24	20	15	5	sp|Q39219|AOX1A_ARATH
		D.long030315.01	22	11	14	8	6	sp|Q8LEE7|AOX3_ARATH
		D.long030316.01	37	63	30	16	14	sp|F4KD71|DUR3_ARATH
		D.long030317.01	7	125	12	4	8	sp|Q7G6K7|FH3_ORYSJ
		D.long030318.01	0	0	11	3	8	unknown
		D.long030320.01	8	43	19	5	14	sp|Q9C6S1|FH14_ARATH
		D.long030321.01	6	16	6	6	0	unknown
		D.long030322.01	26	17	0	0	0	sp|O04066|ACBP_RICCO
		D.long030323.01	6	0	13	3	10	sp|Q9C801|MOS2_ARATH
		D.long030324.01	15	320	26	10	16	sp|Q9FT73|RECQ2_ARATH
		D.long030325.01	21	23	6	5	1	sp|Q9FF09|PIA1_ARATH
		D.long030326.01	4	0	32	9	23	sp|Q8GX29|SKI25_ARATH
		D.long030327.01	0	27	12	2	10	unknown
		D.long030328.01	5	0	2	0	2	sp|O80450|TGT3B_ARATH
		D.long030329.01	20	110	12	2	10	unknown

Trait	Chromosome	Gene id	Promoter	Intron	CDS	Synonymous	Nonsynonymous	Swiss.port.anno
**Seed Weight**	chr12	D.long038987.01	6	1	3	2	1	sp|G9M9M0|F6H11_IPOBA
		D.long038988.01	15	0	11	3	8	sp|Q94HW2|POLR1_ARATH
		D.long038989.01	11	67	28	5	23	sp|Q94HW2|POLR1_ARATH
		D.long038990.01	9	0	16	8	8	sp|Q9ZSP6|AIR1L_ARATH
		D.long038991.01	0	34	11	3	8	unknown
		D.long038992.01	40	62	68	20	48	unknown
		D.long038993.01	2	0	6	2	4	sp|Q9LMU2|KTI5_ARATH
		D.long038994.01	5	0	4	1	3	sp|Q9LMU2|KTI5_ARATH
		D.long038995.01	14	0	22	5	17	sp|P10978|POLX_TOBAC
		D.long038996.01	12	30	42	7	35	unknown
		D.long038997.01	4	0	9	1	8	unknown
		D.long038998.01	28	13	64	22	42	unknown
		D.long038999.01	31	72	60	22	38	unknown
		D.long039000.01	1	0	1	0	1	sp|Q9LMU2|KTI5_ARATH
		D.long039001.01	15	5	21	5	16	unknown
		D.long039002.01	1	9	0	0	0	sp|P32765|ASP_THECC
		D.long039003.01	42	0	59	14	45	sp|P10978|POLX_TOBAC
		D.long039004.01	63	105	197	75	122	unknown
		D.long039005.01	1	0	0	0	0	sp|Q9LMU2|KTI5_ARATH
	chr3	D.long019821.01	11	46	8	3	5	sp|Q6NQ81|PP304_ARATH
		D.long019822.01	5	5	4	2	2	sp|Q8LPS2|ACD6_ARATH
		D.long019823.01	13	33	19	9	10	sp|F4JW79|RDM3_ARATH
		D.long019824.01	10	98	16	9	7	unknown
		D.long019825.01	49	10	90	72	18	sp|Q8LPS2|ACD6_ARATH
		D.long019826.01	9	50	62	18	44	unknown
		D.long019827.01	11	12	4	3	1	sp|Q6YUU5|MDR_ORYSJ
		D.long019828.01	10	0	16	10	6	sp|O48915|NDR1_ARATH
		D.long019829.01	1	2	1	0	1	sp|O49561|G2OX8_ARATH
		D.long019830.01	1	0	1	0	1	sp|A3BDI8|SAP8_ORYSJ
		D.long019831.01	28	77	2	1	1	unknown
		D.long019832.01	11	0	18	18	0	sp|Q8H159|UBQ10_ARATH
		D.long019833.01	19	0	31	31	0	unknown
		D.long019834.01	28	67	11	1	10	unknown
		D.long019835.01	0	4	1	0	1	sp|P41568|SUI11_ARATH
		D.long019836.01	1	12	2	2	0	sp|O22059|CPC_ARATH
		D.long019837.01	8	33	24	14	10	sp|O23787|THI4_CITSI
		D.long019838.01	8	0	19	7	12	unknown
		D.long019839.01	38	49	49	20	29	sp|O23787|THI4_CITSI
		D.long019840.01	63	9	32	15	17	sp|Q8LPS2|ACD6_ARATH
		D.long019841.01	12	29	13	3	10	sp|Q94C53|DDRGK_ARATH
		D.long019842.01	10	23	12	12	0	sp|Q9LJU0|CBL10_ARATH
		D.long019843.01	7	7	52	17	35	sp|Q6JN46|EIX2_SOLLC
		D.long019844.01	9	5	25	9	16	sp|Q6JN46|EIX2_SOLLC
		D.long019845.01	8	36	2	1	1	unknown
		D.long019846.01	53	138	107	42	65	sp|Q6JN47|EIX1_SOLLC
		D.long019847.01	12	140	6	3	3	unknown
		D.long019848.01	8	8	9	4	5	sp|Q9SK27|ENL1_ARATH
		D.long019849.01	0	1	1	1	0	sp|Q9LJU2|PDI53_ARATH
	chr6	D.long024180.01	25	426	21	9	12	unknown
		D.long024181.01	35	58	5	1	4	unknown
		D.long024182.01	30	75	6	5	1	sp|Q0WTY4|VPS2B_ARATH
		D.long024183.01	5	382	3	0	3	sp|P94077|LSD1_ARATH
		D.long024184.01	8	16	5	3	2	sp|B9GIE4|CSPL5_POPTR
		D.long024185.01	7	131	92	42	50	sp|Q8GUI6|JMJ14_ARATH
		D.long024186.01	21	0	24	11	13	sp|P0DH64|Y4891_SELML
		D.long024187.01	49	147	23	7	16	sp|Q9SPE5|SNAG_ARATH
		D.long024188.01	11	150	62	15	47	sp|Q9FI12|SBT23_ARATH
		D.long024189.01	24	114	18	6	12	sp|Q9FI13|RPI4_ARATH
		D.long024190.01	25	0	12	8	4	unknown
		D.long024191.01	12	195	14	4	10	sp|Q9SA77|ARAE1_ARATH
		D.long024192.01	7	57	22	14	8	sp|Q9LI74|CHUP1_ARATH
		D.long024193.01	17	0	2	0	2	sp|Q941W1|G1L7_ORYSJ
		D.long024194.01	5	0	5	2	3	sp|Q9FGH2|LSH5_ARATH
		D.long024130.01	14	0	104	39	65	sp|Q39214|RPM1_ARATH
		D.long024131.01	9	129	52	12	40	sp|V9M2S5|RPV1_VITRO
		D.long024132.01	35	23	48	16	32	sp|Q9LQ55|DRP2B_ARATH
		D.long024133.01	16	4	13	2	11	sp|O65440|BAME3_ARATH
		D.long024134.01	1	24	14	2	12	unknown
		D.long024135.01	0	34	21	5	16	unknown
		D.long024135.02	9	0	0	0	0	sp|Q5D869|NRPE1_ARATH
		D.long024136.01	9	483	178	49	129	sp|Q9FI14|TAO1_ARATH
		D.long024137.01	13	159	33	11	22	sp|Q9SE83|DRP2A_ARATH
		D.long024138.01	13	52	4	3	1	sp|Q9M565|TAF11_ARATH
		D.long024139.01	4	9	0	0	0	unknown
		D.long024140.01	0	0	2	0	2	unknown
		D.long024141.01	9	51	6	6	0	sp|Q8LGI3|ARC2A_ARATH
		D.long024142.01	28	7	9	6	3	unknown
		D.long024143.01	0	22	96	33	63	unknown
		D.long024143.02	26	0	0	0	0	unknown
		D.long024144.01	10	44	26	13	13	sp|F4JUU5|S2P_ARATH
		D.long024145.01	1	25	8	5	3	unknown
		D.long024146.01	7	51	21	8	13	unknown
		D.long024147.01	11	55	6	1	5	sp|Q9FIW4|BGL42_ARATH
		D.long024148.01	38	75	16	3	13	sp|Q9FIW4|BGL42_ARATH
		D.long024149.01	18	164	20	12	8	sp|Q9FIW4|BGL42_ARATH
		D.long024150.01	3	81	27	8	19	sp|Q8W1Y0|SSC14_ARATH
		D.long024151.01	7	62	25	7	18	unknown
		D.long024152.01	30	321	38	16	22	sp|Q9FFF4|ILVH1_ARATH
		D.long024153.01	10	4	19	11	8	sp|Q9ASS4|Y5838_ARATH
		D.long024154.01	10	7	0	0	0	unknown
		D.long024155.01	46	34	18	7	11	sp|Q9SLN8|DBR_TOBAC
		D.long024156.01	21	187	76	17	59	sp|Q84W55|IP5PF_ARATH
		D.long024157.01	27	3	4	0	4	unknown
		D.long024158.01	16	32	21	5	16	sp|O82497|DGP2_ARATH

Trait	Chromosome	Gene id	Promoter	Intron	CDS	Synonymous	Nonsynonymous	Swiss.port.anno
**Total Soluble Solid**	chr14	D.long029570.01	3	0	4	2	2	sp|Q2QVG9|CLPC2_ORYSJ
		D.long029571.01	5	17	12	3	9	sp|Q9FI56|CLPC1_ARATH
		D.long029572.01	5	4	7	3	4	unknown
		D.long029573.01	3	0	1	1	0	unknown
		D.long029574.01	4	1	23	9	14	sp|Q9LW32|PP258_ARATH
		D.long029575.01	14	4	6	1	5	sp|Q66GR8|NET3A_ARATH
		D.long029576.01	22	29	11	8	3	sp|Q9FMU6|MPCP3_ARATH
		D.long029577.01	22	36	7	2	5	sp|Q9SDS7|VATC_ARATH
		D.long029578.01	2	20	4	1	3	sp|Q9LW31|FUS3_ARATH
		D.long029579.01	12	19	4	3	1	sp|Q9LSZ9|LCB2A_ARATH
		D.long029580.01	6	0	1	0	1	unknown
		D.long029581.01	3	10	4	4	0	sp|Q9LW29|AFB2_ARATH
		D.long029582.01	6	15	4	0	4	unknown
		D.long029583.01	7	0	2	0	2	sp|Q9LYV6|GEML5_ARATH
		D.long029584.01	7	15	1	1	0	unknown
		D.long029585.01	3	112	6	1	5	unknown
		D.long029586.01	6	3	3	0	3	sp|O22800|COL14_ARATH
		D.long029587.01	12	28	15	6	9	unknown
		D.long029588.01	5	10	1	1	0	sp|B9GFG6|CSPLF_POPTR
		D.long029589.01	31	89	23	12	11	sp|Q9LW26|Y3684_ARATH
		D.long029590.01	6	66	13	3	10	sp|Q9LW26|Y3684_ARATH
		D.long029591.01	20	77	20	6	14	sp|Q9LW26|Y3684_ARATH
		D.long029592.01	8	176	27	20	7	sp|Q9LW26|Y3684_ARATH
		D.long029593.01	8	58	14	5	9	sp|Q9LW26|Y3684_ARATH
		D.long029594.01	2	0	1	0	1	sp|Q9FFX4|KNU_ARATH
		D.long029595.01	3	9	4	3	1	unknown
		D.long029596.01	5	62	45	44	1	unknown
		D.long029597.01	9	0	0	0	0	sp|Q9LW20|SKL1_ARATH
		D.long029597.02	0	5	1	0	1	sp|Q9LW20|SKL1_ARATH
		D.long029598.01	0	6	3	1	2	sp|O22799|Y2349_ARATH
		D.long029599.01	2	19	5	2	3	unknown
		D.long029600.01	2	1	0	0	0	sp|Q9FY93|NAC83_ARATH
		D.long029601.01	7	16	7	4	3	sp|Q9FLM1|GDPD2_ARATH
		D.long029602.01	0	12	8	3	5	sp|Q8GY79|DRB5_ARATH
		D.long029603.01	1	16	3	2	1	sp|Q0WQK2|ZDHC9_ARATH
		D.long029604.01	4	13	6	1	5	sp|Q9FE20|PBS1_ARATH
		D.long029605.01	3	3	2	2	0	unknown
		D.long029606.01	2	0	6	4	2	sp|Q9FGH9|E70B1_ARATH
		D.long029607.01	0	2	1	0	1	unknown
		D.long029608.01	3	0	0	0	0	sp|Q8LF89|GRXC8_ARATH
		D.long029609.01	1	9	0	0	0	sp|Q9LSD8|MUB4_ARATH
		D.long029610.01	2	0	4	3	1	sp|A0A145P7T2|RAM1_LOTJA
		D.long029611.01	4	3	4	3	1	sp|Q6NMJ2|OLE96_ARATH
		D.long029612.01	8	5	10	4	6	sp|Q94CD1|HHT1_ARATH
		D.long029613.01	4	34	2	2	0	sp|Q9LSD6|ARP2_ARATH
		D.long029614.01	10	0	0	0	0	sp|P0DKK7|RL7A2_ORYSJ
	chr3	D.long019020.01	36	184	18	5	13	sp|Q9SYM0|VTE6_ARATH
		D.long019021.01	15	54	9	6	3	sp|Q9SYL9|RK13_ARATH
		D.long019022.01	15	125	24	13	11	sp|Q9ZV89|TBL42_ARATH
		D.long019023.01	1	104	22	5	17	sp|Q8VY22|TBL38_ARATH
		D.long019024.01	12	2	10	2	8	unknown
		D.long019025.01	20	10	44	15	29	unknown
		D.long019026.01	4	0	24	10	14	unknown
		D.long019027.01	5	79	17	6	11	sp|Q8VY22|TBL38_ARATH
		D.long019028.01	9	62	15	7	8	sp|Q8VY22|TBL38_ARATH
		D.long019029.01	33	81	55	23	32	unknown
		D.long019030.01	32	159	17	6	11	sp|F4IWA8|TBL41_ARATH
		D.long019031.01	13	227	29	12	17	unknown
		D.long019032.01	0	8	8	4	4	unknown
		D.long019033.01	17	24	43	12	31	unknown
		D.long019034.01	23	52	33	10	23	unknown
		D.long019035.01	2	25	105	45	60	unknown
		D.long019036.01	19	93	50	11	39	unknown
		D.long019801.01	10	26	4	2	2	sp|Q9FH37|ILR3_ARATH
		D.long019802.01	7	0	9	3	6	unknown
		D.long019803.01	6	49	23	17	6	sp|Q9FH36|GAUTC_ARATH
		D.long019804.01	3	1	30	12	18	sp|Q9T048|DRL27_ARATH
		D.long019805.01	6	0	6	3	3	unknown
		D.long019806.01	8	0	13	4	9	unknown
		D.long019807.01	0	0	21	10	11	sp|Q9T048|DRL27_ARATH
		D.long019808.01	7	0	6	0	6	sp|Q9LMP6|DRL3_ARATH
		D.long019810.01	4	12	3	1	2	sp|Q0WVV0|PPR31_ARATH
		D.long019811.01	2	9	13	2	11	sp|Q9T048|DRL27_ARATH
		D.long019812.01	14	14	29	13	16	sp|Q9T048|DRL27_ARATH
		D.long019813.01	31	4	49	16	33	sp|Q9T048|DRL27_ARATH
		D.long019814.01	3	3	19	6	13	sp|Q9T048|DRL27_ARATH
		D.long019816.01		11	18	5	13	sp|Q9T048|DRL27_ARATH
		D.long019817.01	4	38	20	2	18	sp|Q9FH32|AT18F_ARATH
		D.long019818.01	8	111	36	26	10	unknown
		D.long019819.01	27	42	9	1	8	sp|Q6ZJX0|FIE2_ORYSJ
		D.long019820.01	21	0	21	11	10	unknown
		D.long019821.01	11	46	10	4	6	sp|Q6NQ81|PP304_ARATH
		D.long019822.01	5	5	4	2	2	sp|Q8LPS2|ACD6_ARATH
		D.long019823.01	13	33	19	9	10	sp|F4JW79|RDM3_ARATH
		D.long019824.01	10	98	16	9	7	unknown
		D.long019825.01	49	10	90	72	18	sp|Q8LPS2|ACD6_ARATH
		D.long019826.01	9	50	62	18	44	unknown
		D.long019827.01	11	12	4	3	1	sp|Q6YUU5|MDR_ORYSJ
		D.long019828.01	10	0	16	10	6	sp|O48915|NDR1_ARATH
	chr9	D.long022501.01	1	0	62	27	35	sp|Q7X8C5|WAKLB_ARATH
		D.long022502.01	40	178	81	13	68	sp|Q9SA25|WAKLG_ARATH
		D.long022503.01	13	7	21	7	14	unknown
		D.long022504.01	36	150	139	41	98	sp|Q8RY17|WAKLI_ARATH
		D.long022505.01	26	12	89	29	60	unknown
		D.long022506.01	2	5	79	33	46	unknown
		D.long022507.01	38	18	42	11	31	unknown
		D.long022508.01	1	7	85	36	49	unknown
		D.long022509.01	8	19	69	26	43	unknown
		D.long022510.01	3	0	67	28	39	unknown
		D.long022511.01	22	13	36	11	25	unknown
		D.long022512.01	52	0	213	70	143	sp|Q9SIT7|PP151_ARATH
		D.long022513.01	33	63	38	12	26	unknown
		D.long022514.01	32	118	78	43	35	sp|Q9S9M5|WAKLA_ARATH
		D.long022515.01	30	0	6	6	0	sp|Q9ZT94|POLR2_ARATH
		D.long022516.01	2	29	28	9	19	unknown
		D.long022517.01	28	60	28	8	20	sp|Q9C7N4|GDL15_ARATH
		D.long022518.01	16	48	68	21	47	sp|Q9LTE3|HIP12_ARATH
		D.long022519.01	1	2	2	2	0	sp|Q9ZT94|POLR2_ARATH
		D.long022520.01	13	11	24	8	16	unknown
		D.long022521.01	19	28	1	0	1	sp|Q9C7N4|GDL15_ARATH

Table S11. SNPs at three genes related to TSS and seed weight .

Gene ID: D. long019821

ID	gene element	Reference	Alternate	BYZ-FJ	CK-FJ	HBP-FJ	FY-FJ	QZB-FJ	JYW-FJ	GSEH-GD	HLGY-GD	YTB-FJ	LQB-FJ	LY-GD	JL-FJ	WLL-FJ	SSCR-GD	JY-GD	SX-GD	HH-GD	HDGY-GD
chr3:18379687	CDS	A	T	0 0	0 0	0 0	0 0	0 0	0 0	0 0	0 0	0 0	0 0	0 0	0 0	0 0	0 1	0 1	0 1	0 1	0 1
chr3:18379727	CDS	A	G	0 0	0 0	0 0	0 0	0 0	0 0	0 0	0 0	0 0	0 0	0 0	0 0	0 0	0 1	0 1	0 1	0 1	0 1
chr3:18380152	Splice	G	C	0 0	0 0	0 0	0 0	0 0	0 0	0 0	0 0	0 0	0 0	0 0	0 0	0 0	0 1	0 1	0 1	0 1	0 1
chr3:18380851	CDS	A	G	0 0	0 0	0 0	0 0	0 0	0 0	0 0	0 0	0 0	0 0	0 0	0 0	0 0	0 1	0 1	0 1	0 1	0 1
chr3:18380866	CDS	A	G	0 0	0 0	0 0	0 0	0 0	0 0	0 0	0 0	0 0	0 0	0 0	0 0	0 0	0 1	0 1	0 1	0 1	0 1
chr3:18380889	Intron	G	A	0 0	0 0	0 0	0 0	0 0	0 0	0 0	0 0	0 0	0 0	0 0	0 0	0 0	0 1	0 1	0 1	0 1	0 1
chr3:18380915	Intron	A	T	0 0	0 0	0 0	0 0	0 0	0 0	0 0	0 0	0 0	0 0	0 0	0 0	0 0	0 1	0 1	0 1	0 1	0 1
chr3:18381202	Intron	C	A	0 0	0 0	0 0	0 0	0 0	0 0	0 0	0 0	0 0	0 0	0 0	0 0	0 0	0 1	0 1	0 1	0 1	0 1
chr3:18381237	Intron	C	A	0 0	0 0	0 0	0 0	0 0	0 0	0 0	0 0	0 0	0 0	0 0	0 0	0 0	0 1	0 1	0 1	0 1	0 1
chr3:18381266	Intron	T	A	0 0	0 0	0 0	0 0	0 0	0 0	0 0	0 0	0 0	0 0	0 0	0 0	0 0	0 1	0 1	0 1	0 1	0 1
chr3:18381657	Intron	G	T	0 0	0 0	0 0	0 0	0 0	0 0	0 0	0 0	0 0	0 0	0 0	0 0	0 0	0 1	0 1	0 1	0 1	0 1
chr3:18381872	Intron	T	C	0 0	0 0	0 0	0 0	0 0	0 0	0 0	0 0	0 0	0 0	0 0	0 0	0 0	0 1	0 1	0 1	0 1	0 1
chr3:18382077	Intron	A	C	0 0	0 0	0 0	0 0	0 0	0 0	0 0	0 0	0 0	0 0	0 0	0 0	0 0	0 1	0 1	0 1	0 1	**1 1**
chr3:18382363	Intron	T	A	0 0	0 0	0 0	0 0	0 0	0 0	0 0	0 0	0 0	0 0	0 0	0 0	0 0	0 1	0 1	0 1	0 1	**1 1**
chr3:18382917	Intron	G	C	0 0	0 0	0 0	0 0	0 0	0 0	0 0	0 0	0 0	0 0	0 0	0 0	0 0	0 1	0 1	0 1	0 1	**1 1**
chr3:18383098	Intron	T	A	0 0	0 0	0 0	0 0	0 0	0 0	0 0	0 0	0 0	0 0	0 0	0 0	0 0	0 1	0 1	0 1	0 1	0 1
chr3:18383184	Intron	T	C	0 0	0 0	0 0	0 0	0 0	0 0	0 0	0 0	0 0	0 0	0 0	0 0	0 0	0 1	0 1	0 1	0 1	0 1
chr3:18383483	Intron	C	T	0 0	0 0	0 0	0 0	0 0	0 0	0 0	0 0	0 0	0 0	0 0	0 0	0 0	0 1	0 1	0 1	0 1	0 1
chr3:18383702	Intron	G	A	0 0	0 0	0 0	0 0	0 0	0 0	0 0	0 0	0 0	0 0	0 0	0 0	0 0	0 1	0 1	0 1	0 1	0 1
chr3:18383849	Intron	C	T	0 0	0 0	0 0	0 0	0 0	0 0	0 0	0 0	0 0	0 0	0 0	0 0	0 0	0 1	0 1	0 1	0 1	0 1
chr3:18383970	Intron	A	C	0 0	0 0	0 0	0 0	0 0	0 0	0 0	0 0	0 0	0 0	0 0	0 0	0 0	0 1	0 1	0 1	0 1	0 1
chr3:18384029	Intron	A	G	0 0	0 0	0 0	0 0	0 0	0 0	0 0	0 0	0 0	0 0	0 0	0 0	0 0	0 1	0 1	0 1	0 1	0 1
chr3:18384183	Intron	G	C	0 0	0 0	0 0	0 0	0 0	0 0	0 0	0 0	0 0	0 0	0 0	0 0	0 0	0 1	0 1	0 1	0 1	**1 1**
chr3:18384713	Intron	G	A	0 0	0 0	0 0	0 0	0 0	0 0	0 0	0 0	0 0	0 0	0 0	0 0	0 0	0 1	0 1	0 1	0 0	0 1
chr3:18384919	Intron	G	A	0 0	0 0	0 0	0 0	0 0	0 0	0 0	0 0	0 0	0 0	0 0	0 0	0 0	0 1	0 1	0 1	0 1	0 1
chr3:18385255	Intron	T	C	0 0	0 0	0 0	0 0	0 0	0 0	0 0	0 0	0 0	0 0	0 0	0 0	0 0	0 1	0 1	0 1	0 1	**1 1**
chr3:18385334	Intron	G	A	0 0	0 0	0 0	0 0	0 0	0 0	0 0	0 0	0 0	0 0	0 0	0 0	0 0	0 1	0 1	0 1	0 1	0 1
chr3:18385628	Intron	A	G	0 0	0 0	0 0	0 0	0 0	0 0	0 0	0 0	0 0	0 0	0 0	0 0	0 0	0 1	0 1	0 1	0 1	0 1
chr3:18385671	Intron	C	T	0 0	0 0	0 0	0 0	0 0	0 0	0 0	0 0	0 0	0 0	0 0	0 0	0 0	0 1	0 1	0 1	0 1	**1 1**
chr3:18385705	Intron	G	T	0 0	0 0	0 0	0 0	0 0	0 0	0 0	0 0	0 0	0 0	0 0	0 0	0 0	0 1	0 1	0 1	0 1	0 1
chr3:18385708	Intron	C	A	0 0	0 0	0 0	0 0	0 0	0 0	0 0	0 0	0 0	0 0	0 0	0 0	0 0	0 1	0 1	0 1	0 1	**1 1**
chr3:18385893	Intron	C	G	0 0	0 0	0 0	0 0	0 0	0 0	0 0	0 0	0 0	0 0	0 0	0 0	0 0	0 1	0 1	0 1	0 1	**1 1**
chr3:18386236	Intron	T	C	0 0	0 0	0 0	0 0	0 0	0 0	0 0	0 0	0 0	0 0	0 0	0 0	0 0	0 1	0 1	0 1	0 1	**1 1**
chr3:18386269	Intron	G	A	0 0	0 0	0 0	0 0	0 0	0 0	0 0	0 0	0 0	0 0	0 0	0 0	0 0	0 1	0 1	0 1	0 1	0 1
chr3:18386527	Intron	C	T	0 0	0 0	0 0	0 0	0 0	0 0	0 0	0 0	0 0	0 0	0 0	0 0	0 0	0 1	0 1	0 1	0 1	0 1
chr3:18386555	Intron	A	G	0 0	0 0	0 0	0 0	0 0	0 0	0 0	0 0	0 0	0 0	0 0	0 0	0 0	0 1	0 1	0 1	0 1	**1 1**
chr3:18386896	Intron	G	T	0 0	0 0	0 0	0 0	0 0	0 0	0 0	0 0	0 0	0 0	0 0	0 0	0 0	0 1	0 1	0 1	0 1	0 1
chr3:18386914	Intron	T	G	0 0	0 0	0 0	0 0	0 0	0 0	0 0	0 0	0 0	0 0	0 0	0 0	0 0	0 1	0 1	0 1	0 1	0 1
chr3:18387476	Intron	A	C	0 0	0 0	0 0	0 0	0 0	0 0	0 0	0 0	0 0	0 0	0 0	0 0	0 0	0 1	0 1	0 1	0 1	**1 1**
chr3:18387539	Intron	A	G	0 0	0 0	0 0	0 0	0 0	0 0	0 0	0 0	0 0	0 0	0 0	0 0	0 0	0 1	0 1	0 1	0 1	0 1
chr3:18387641	CDS	C	T	0 0	0 0	0 0	0 0	0 0	0 0	0 0	0 0	0 0	0 0	0 0	0 0	0 0	0 1	0 1	0 1	0 1	**1 1**
chr3:18387755	CDS	T	C	0 0	0 0	0 0	0 0	0 0	0 0	0 0	0 0	0 0	0 0	0 0	0 0	0 0	0 1	0 1	0 1	0 1	0 1
chr3:18388087	CDS	G	A	0 0	0 0	0 0	0 0	0 0	0 0	0 0	0 0	0 0	0 0	0 0	0 0	0 0	0 1	0 1	0 1	0 1	0 1
chr3:18388347	Intron	T	C	0 0	0 0	0 0	0 0	0 0	0 0	0 0	0 0	0 0	0 0	0 0	0 0	0 0	0 1	0 1	0 1	0 1	**1 1**
chr3:18388400	Intron	G	A	0 0	0 0	0 0	0 0	0 0	0 0	0 0	0 0	0 0	0 0	0 0	0 0	0 0	0 1	0 1	0 1	0 1	0 1
chr3:18388487	Intron	T	A	0 0	0 0	0 0	0 0	0 0	0 0	0 0	0 0	0 0	0 0	0 0	0 0	0 0	0 1	0 1	0 1	0 1	**1 1**
chr3:18388518	Intron	T	C	0 0	0 0	0 0	0 0	0 0	0 0	0 0	0 0	0 0	0 0	0 0	0 0	0 0	0 1	0 1	0 1	0 1	**1 1**
chr3:18388579	Intron	A	G	0 0	0 0	0 0	0 0	0 0	0 0	0 0	0 0	0 0	0 0	0 0	0 0	0 0	0 1	0 1	0 1	0 1	0 1
chr3:18388617	Intron	T	C	0 0	0 0	0 0	0 0	0 0	0 0	0 0	0 0	0 0	0 0	0 0	0 0	0 0	0 1	0 1	0 1	0 1	**1 1**
chr3:18388628	Intron	A	G	0 0	0 0	0 0	0 0	0 0	0 0	0 0	0 0	0 0	0 0	0 0	0 0	0 0	0 1	0 1	0 1	0 1	**1 1**
chr3:18388925	Intron	C	T	0 0	0 0	0 0	0 0	0 0	0 0	0 0	0 0	0 0	0 0	0 0	0 0	0 0	0 1	0 1	0 1	0 1	0 1
chr3:18389085	Intron	A	G	0 0	0 0	0 0	0 0	0 0	0 0	0 0	0 0	0 0	0 0	0 0	0 0	0 0	0 0	0 0	0 0	0 0	0 1
chr3:18389103	Intron	C	A	0 0	0 0	0 0	0 0	0 0	0 0	0 0	0 0	0 0	0 0	0 0	0 0	0 0	0 1	0 1	0 1	0 0	0 1
chr3:18389111	Intron	G	T	0 0	0 0	0 0	0 0	0 0	0 0	0 0	0 0	0 0	0 0	0 0	0 0	0 0	0 1	0 1	0 1	0 1	0 1
chr3:18389180	CDS	A	G	0 0	0 0	0 0	0 0	0 0	0 0	0 0	0 0	0 0	0 0	0 0	0 0	0 0	0 0	0 0	0 0	0 1	**1 1**
chr3:18389234	CDS	C	G	0 0	0 0	0 0	0 0	0 0	0 0	0 0	0 0	0 0	0 0	0 0	0 0	0 0	0 1	0 1	0 1	0 1	**1 1**
chr3:18389338	CDS	T	C	0 0	0 0	0 0	0 0	0 0	0 0	0 0	0 0	0 0	0 0	0 0	0 0	0 0	0 1	0 1	0 1	0 1	**1 1**
chr3:18389374	Promoter	C	G	0 0	0 0	0 0	0 0	0 0	0 0	0 0	0 0	0 0	0 0	0 0	0 0	0 0	0 1	0 1	0 1	0 1	**1 1**
chr3:18389426	Promoter	T	C	0 0	0 0	0 0	0 0	0 0	0 0	0 0	0 0	0 0	0 0	0 0	0 0	0 0	0 1	0 1	0 1	0 1	0 1
chr3:18389437	Promoter	A	G	0 0	0 0	0 0	0 0	0 0	0 0	0 0	0 0	0 0	0 0	0 0	0 0	0 0	0 1	0 1	0 1	0 1	0 1
chr3:18389461	Promoter	C	G	0 0	0 0	0 0	0 0	0 0	0 0	0 0	0 0	0 0	0 0	0 0	0 0	0 0	0 1	0 1	0 1	0 1	0 1
chr3:18389498	Promoter	T	C	0 0	0 0	0 0	0 0	0 0	0 0	0 0	0 0	0 0	0 0	0 0	0 0	0 0	0 1	0 1	0 1	0 1	0 1
chr3:18389556	Promoter	C	T	0 0	0 0	0 0	0 0	0 0	0 0	0 0	0 0	0 0	0 0	0 0	0 0	0 0	0 1	0 1	0 1	0 1	0 1
chr3:18389593	Promoter	T	C	0 0	0 0	0 0	0 0	0 0	0 0	0 0	0 0	0 0	0 0	0 0	0 0	0 0	0 1	0 1	0 1	0 1	0 1
chr3:18389601	Promoter	G	A	0 0	0 0	0 0	0 0	0 0	0 0	0 0	0 0	0 0	0 0	0 0	0 0	0 0	0 1	0 1	0 1	0 1	0 1
chr3:18389722	Promoter	G	A	0 0	0 0	0 0	0 0	0 0	0 0	0 0	0 0	0 0	0 0	0 0	0 0	0 0	0 1	0 1	0 1	0 1	0 1
chr3:18389802	Promoter	G	T	0 0	0 0	0 0	0 0	0 0	0 0	0 0	0 0	0 0	0 0	0 0	0 0	0 0	0 1	0 1	0 1	0 1	**1 1**
chr3:18389834	Promoter	T	G	0 0	0 0	0 0	0 0	0 0	0 0	0 0	0 0	0 0	0 0	0 0	0 0	0 0	0 1	0 1	0 1	0 1	0 1

Gene ID: D. long019822

ID	gene element	Reference	Alternate	BYZ-FJ	CK-FJ	HBP-FJ	FY-FJ	QZB-FJ	JYW-FJ	GSEH-GD	HLGY-GD	YTB-FJ	LQB-FJ	LY-GD	JL-FJ	WLL-FJ	SSCR-GD	JY-GD	SX-GD	HH-GD	HDGY-GD
chr3:18391540	CDS	G	A	0_0	0_0	0_0	0_0	0_0	0_0	0_0	0_0	0_0	0_0	0_0	0_0	0_0	0_1	0_1	0_1	0_1	0_1
chr3:18391690	CDS	G	A	0_0	0_0	0_0	0_0	0_0	0_0	0_0	0_0	0_0	0_0	0_0	0_0	0_0	0_1	0_1	0_1	0_1	0_1
chr3:18392224	Intron	T	C	0_0	0_0	0_0	0_0	0_0	0_0	0_0	0_0	0_0	0_0	0_0	0_0	0_0	0_1	0_1	0_1	0_1	**1_1**
chr3:18392235	Intron	T	C	0_0	0_0	0_0	0_0	0_0	0_0	0_0	0_0	0_0	0_0	0_0	0_0	0_0	0_1	0_1	0_1	0_1	0_1
chr3:18392382	Intron	T	C	0_0	0_0	0_0	0_0	0_0	0_0	0_0	0_0	0_0	0_0	0_0	0_0	0_0	0_1	0_1	0_1	0_1	**1_1**
chr3:18392908	CDS	A	G	0_0	0_0	0_0	0_0	0_0	0_0	0_0	0_0	0_0	0_0	0_0	0_0	0_0	0_0	0_0	0_0	0_0	0_1
chr3:18392909	CDS	T	C	0_0	0_0	0_0	0_0	0_0	0_0	0_0	0_0	0_0	0_0	0_0	0_0	0_0	0_0	0_0	0_0	0_0	0_1
chr3:18393109	Intron	T	G	0_0	0_0	0_0	0_0	0_0	0_0	0_0	0_0	0_0	0_0	0_0	0_0	0_0	0_0	0_0	0_0	0_0	0_1
chr3:18393110	Intron	C	T	0_0	0_0	0_0	0_0	0_0	0_0	0_0	0_0	0_0	0_0	0_0	0_0	0_0	0_0	0_0	0_0	0_0	0_1
chr3:18393922	Promoter	C	G	0_0	0_0	0_0	0_0	0_0	0_0	0_0	0_0	0_0	0_0	0_0	0_0	0_0	0_0	0_0	0_0	0_0	0_1
chr3:18393939	Promoter	T	A	0_0	0_0	0_0	0_0	0_0	0_0	0_0	0_0	0_0	0_0	0_0	0_0	0_0	0_0	0_0	0_0	0_0	0_1
chr3:18393961	Promoter	A	G	0_0	0_0	0_0	0_0	0_0	0_0	0_0	0_0	0_0	0_0	0_0	0_0	0_0	0_1	0_1	0_1	0_0	**1_1**
chr3:18394023	Promoter	A	T	0_0	0_0	0_0	0_0	0_0	0_0	0_0	0_0	0_0	0_0	0_0	0_0	0_0	0_1	0_1	0_1	0_0	**1_1**
chr3:18394051	Promoter	G	A	0_0	0_0	0_0	0_0	0_0	0_0	0_0	0_0	0_0	0_0	0_0	0_0	0_0	0_1	0_1	0_1	0_0	**1_1**

Gene ID: D. long019823

ID	gene element	Reference	Alternate	BYZ-FJ	CK-FJ	HBP-FJ	FY-FJ	QZB-FJ	JYW-FJ	GSEH-GD	HLGY-GD	YTB-FJ	LQB-FJ	LY-GD	JL-FJ	WLL-FJ	SSCR-GD	JY-GD	SX-GD	HH-GD	HDGY-GD
chr3:18405540	Promoter	C	T	0 0	0 0	0 0	0 0	0 0	0 0	0 0	0 0	0 0	0 0	0 0	0 0	0 0	0 1	0 1	0 1	0 0	0 1
chr3:18405577	Promoter	T	C	0 0	0 0	0 0	0 0	0 0	0 0	0 0	0 0	0 0	0 0	0 0	0 0	0 0	0 0	0 0	0 0	0 0	0 0
chr3:18405608	Promoter	A	G	0 0	0 0	0 0	0 0	0 0	0 0	0 0	0 0	0 0	0 0	0 0	0 0	0 0	0 0	0 0	0 0	0 0	0 0
chr3:18405618	Promoter	T	C	0 0	0 0	0 0	0 0	0 0	0 0	0 0	0 0	0 0	0 0	0 0	0 0	0 0	0 0	0 0	0 0	0 0	0 0
chr3:18405620	Promoter	C	A	0 0	0 0	0 0	0 0	0 0	0 0	0 0	0 0	0 0	0 0	0 0	0 0	0 0	0 1	0 1	0 1	0 0	0 1
chr3:18405629	Promoter	T	C	0 0	0 0	0 0	0 0	0 0	0 0	0 0	0 0	0 0	0 0	0 0	0 0	0 0	0 0	0 0	0 0	0 0	0 0
chr3:18405638	Promoter	C	T	0 0	0 0	0 0	0 0	0 0	0 0	0 0	0 0	0 0	0 0	0 0	0 0	0 0	0 1	0 1	0 1	0 0	**1 1**
chr3:18405646	Promoter	T	C	0 0	0 0	0 0	0 0	0 0	0 0	0 0	0 0	0 0	0 0	0 0	0 0	0 0	0 1	0 1	0 1	0 0	**1 1**
chr3:18405671	Promoter	C	T	0 0	0 0	0 0	0 0	0 0	0 0	0 0	0 0	0 0	0 0	0 0	0 0	0 0	0 1	0 1	0 1	0 0	0 1
chr3:18405754	Promoter	T	A	0 0	0 0	0 0	0 0	0 0	0 0	0 0	0 0	0 0	0 0	0 0	0 0	0 0	0 0	0 0	0 0	0 0	0 0
chr3:18405780	Promoter	C	T	0 0	0 0	0 0	0 0	0 0	0 0	0 0	0 0	0 0	0 0	0 0	0 0	0 0	0 1	0 1	0 1	0 0	**1 1**
chr3:18405976	Promoter	C	A	0 0	0 0	0 0	0 0	0 0	0 0	0 0	0 0	0 0	0 0	0 0	0 0	0 0	0 0	0 0	0 0	0 0	0 0
chr3:18406084	CDS	T	G	0 0	0 0	0 0	0 0	0 0	0 0	0 0	0 0	0 0	0 0	0 0	0 0	0 0	0 1	0 1	0 1	0 1	**1 1**
chr3:18406198	Intron	G	A	0 0	0 0	0 0	0 0	0 0	0 0	0 0	0 0	0 0	0 0	0 0	0 0	0 0	0 0	0 0	0 0	0 0	0 0
chr3:18406586	Intron	T	C	0 0	0 0	0 0	0 0	0 0	0 0	0 0	0 0	0 0	0 0	0 0	0 0	0 0	0 1	0 1	0 1	0 0	**1 1**
chr3:18406600	Intron	C	T	0 0	0 0	0 0	0 0	0 0	0 0	0 0	0 0	0 0	0 0	0 0	0 0	0 0	0 0	0 0	0 0	0 0	0 0
chr3:18406720	Intron	G	T	0 0	0 0	0 0	0 0	0 1	0 0	0 1	0 0	0 0	0 0	0 0	0 0	0 0	0 1	0 1	**1 1**	0 1	**1 1**
chr3:18406747	Intron	G	T	0 0	0 0	0 0	0 0	0 0	0 0	0 0	0 0	0 0	0 0	0 0	0 0	0 0	0 1	0 1	0 1	0 0	0 1
chr3:18406806	Intron	T	C	0 0	0 0	0 0	0 0	0 0	0 0	0 0	0 0	0 0	0 0	0 0	0 0	0 0	0 1	0 1	0 1	0 0	**1 1**
chr3:18406861	Intron	C	A	0 0	0 0	0 0	0 0	0 0	0 0	0 0	0 0	0 0	0 0	0 0	0 0	0 0	0 1	0 1	0 1	0 0	**1 1**
chr3:18406990	Intron	C	T	0 0	0 0	0 0	0 0	0 0	0 0	0 0	0 0	0 0	0 0	0 0	0 0	0 0	0 1	0 1	0 1	0 0	**1 1**
chr3:18407080	Intron	T	C	0 0	0 0	0 0	0 0	0 0	0 0	0 0	0 0	0 0	0 0	0 0	0 0	0 0	0 1	0 1	0 1	0 0	**1 1**
chr3:18407551	Intron	T	C	0 0	0 0	0 0	0 0	0 0	0 0	0 0	0 0	0 0	0 0	0 0	0 0	0 0	0 1	0 1	0 1	0 0	**1 1**
chr3:18407552	Intron	A	C	0 0	0 0	0 0	0 0	0 0	0 0	0 0	0 0	0 0	0 0	0 0	0 0	0 0	0 1	0 1	0 1	0 0	**1 1**
chr3:18407608	CDS	T	G	0 0	0 0	0 0	0 0	0 0	0 0	0 0	0 0	0 0	0 0	0 0	0 0	0 0	0 1	0 1	0 1	0 0	**1 1**
chr3:18408488	Intron	T	C	0 0	0 0	0 0	0 0	0 0	0 0	0 0	0 0	0 0	0 0	0 0	0 0	0 0	0 1	0 1	0 1	0 0	0 1
chr3:18408525	CDS	A	G	0 0	0 0	0 0	0 0	0 0	0 0	0 0	0 0	0 0	0 0	0 0	0 0	0 0	0 0	0 0	0 0	0 0	0 0
chr3:18408642	Intron	G	T	0 0	0 0	0 0	0 0	0 0	0 0	0 0	0 0	0 0	0 0	0 0	0 0	0 0	0 1	0 1	0 1	0 0	**1 1**
chr3:18408658	Intron	C	G	0 0	0 0	0 0	0 0	0 0	0 0	0 0	0 0	0 0	0 0	0 0	0 0	0 0	0 1	0 1	0 1	0 0	0 1
chr3:18408767	Intron	G	A	0 0	0 0	0 0	0 0	0 0	0 0	0 0	0 0	0 0	0 0	0 0	0 0	0 0	0 1	0 1	0 1	0 0	**1 1**
chr3:18408916	Intron	C	A	0 0	0 0	0 0	0 0	0 0	0 0	0 0	0 0	0 0	0 0	0 0	0 0	0 0	0 1	0 1	0 1	0 0	**1 1**
chr3:18409019	Intron	T	G	0 0	0 0	0 0	0 0	0 0	0 0	0 0	0 0	0 0	0 0	0 0	0 0	0 0	0 1	0 1	0 1	0 0	**1 1**
chr3:18409021	Intron	G	A	0 0	0 0	0 0	0 0	0 0	0 0	0 0	0 0	0 0	0 0	0 0	0 0	0 0	0 1	0 1	0 1	0 0	0 1
chr3:18409088	Intron	G	T	0 0	0 0	0 0	0 0	0 0	0 0	0 0	0 0	0 0	0 0	0 0	0 0	0 0	0 1	0 1	0 1	0 0	**1 1**
chr3:18409249	Intron	A	G	0 0	0 0	0 0	0 0	0 0	0 0	0 0	0 0	0 0	0 0	0 0	0 0	0 0	0 1	0 1	0 1	0 0	0 1
chr3:18410115	CDS	G	A	0 0	0 0	0 0	0 0	0 0	0 0	0 0	0 0	0 0	0 0	0 0	0 0	0 0	0 1	0 1	0 1	0 0	0 1
chr3:18410315	CDS	G	T	0 0	0 0	0 0	0 0	0 0	0 0	0 0	0 0	0 0	0 0	0 0	0 0	0 0	0 1	0 1	0 1	0 0	0 1
chr3:18410317	CDS	T	A	0 0	0 0	0 0	0 0	0 0	0 0	0 0	0 0	0 0	0 0	0 0	0 0	0 0	0 1	0 1	0 1	0 0	**1 1**
chr3:18411040	Intron	G	A	0 0	0 0	0 0	0 0	0 0	0 0	0 0	0 0	0 0	0 0	0 0	0 0	0 0	0 1	0 1	0 1	0 0	**1 1**
chr3:18411139	CDS	G	A	0 0	0 0	0 0	0 0	0 0	0 0	0 0	0 0	0 0	0 0	0 0	0 0	0 0	0 1	0 1	0 1	0 0	**1 1**
chr3:18411493	CDS	G	A	0 0	0 0	0 0	0 0	0 0	0 0	0 0	0 0	0 0	0 0	0 0	0 0	0 0	0 1	0 1	0 1	0 0	**1 1**
chr3:18411774	Intron	C	T	0 0	0 0	0 0	0 0	0 0	0 0	0 0	0 0	0 0	0 0	0 0	0 0	0 0	0 1	0 1	0 1	0 0	0 1
chr3:18412015	Intron	G	T	0 0	0 0	0 0	0 0	0 0	0 0	0 0	0 0	0 0	0 0	0 0	0 0	0 0	0 0	0 0	0 0	0 0	0 0
chr3:18412018	Intron	T	A	0 0	0 0	0 0	0 0	0 0	0 0	0 0	0 0	0 0	0 0	0 0	0 0	0 0	0 1	0 1	0 1	0 0	**1 1**
chr3:18412331	Intron	G	A	0 0	0 0	0 0	0 0	0 0	0 0	0 0	0 0	0 0	0 0	0 0	0 0	0 0	0 1	0 1	0 1	0 0	**1 1**
chr3:18412435	Intron	T	C	0 0	0 0	0 0	0 0	0 0	0 0	0 0	0 0	0 0	0 0	0 0	0 0	0 0	0 0	0 0	0 0	0 0	0 1
chr3:18412436	Intron	G	T	0 0	0 0	0 0	0 0	0 0	0 0	0 0	0 0	0 0	0 0	0 0	0 0	0 0	0 0	0 0	0 0	0 0	0 0
chr3:18412498	Intron	A	G	0 0	0 0	0 0	0 0	0 0	0 0	0 0	0 0	0 0	0 0	0 0	0 0	0 0	0 1	0 1	0 1	0 0	0 1
chr3:18412547	Intron	G	A	0 0	0 0	0 0	0 0	0 0	0 0	0 0	0 0	0 0	0 0	0 0	0 0	0 0	0 1	0 1	0 1	0 0	0 1
chr3:18412689	Intron	T	A	0 0	0 0	0 0	0 0	0 0	0 0	0 0	0 0	0 0	0 0	0 0	0 0	0 0	0 1	0 1	0 1	0 0	0 1
chr3:18412705	Intron	T	A	0 0	0 0	0 0	0 0	0 0	0 0	0 0	0 0	0 0	0 0	0 0	0 0	0 0	0 1	0 1	0 1	0 0	0 1
chr3:18413151	Intron	C	T	0 0	0 0	0 0	0 0	0 0	0 0	0 0	0 0	0 0	0 0	0 0	0 0	0 0	0 1	0 1	0 1	0 0	0 1
chr3:18413585	Intron	T	A	0 0	0 0	0 0	0 0	0 0	0 0	0 0	0 0	0 0	0 0	0 0	0 0	0 0	0 1	0 1	0 1	0 0	**1 1**
chr3:18413981	CDS	T	A	0 0	0 0	0 0	0 0	0 0	0 0	0 0	0 0	0 0	0 0	0 0	0 0	0 0	0 1	0 1	0 1	0 0	0 1
chr3:18414357	CDS	G	A	0 0	0 0	0 0	0 0	0 0	0 0	0 0	0 0	0 0	0 0	0 0	0 0	0 0	0 1	0 1	0 1	0 0	0 1
chr3:18414406	CDS	G	A	0 0	0 0	0 0	0 0	0 0	0 0	0 0	0 0	0 0	0 0	0 0	0 0	0 0	0 1	0 1	0 1	0 0	**1 1**
chr3:18414650	CDS	C	G	0 0	0 0	0 0	0 0	0 0	0 0	0 0	0 0	0 0	0 0	0 0	0 0	0 0	0 1	0 1	0 1	0 0	0 1
chr3:18414980	CDS	T	A	0 0	0 0	0 0	0 0	0 0	0 0	0 0	0 0	0 0	0 0	0 0	0 0	0 0	0 1	0 1	0 1	0 0	**1 1**
chr3:18415364	CDS	C	A	0 0	0 0	0 0	0 0	0 0	0 0	0 0	0 0	0 0	0 0	0 0	0 0	0 0	0 1	0 1	0 1	0 0	0 1
chr3:18415495	CDS	G	C	0 0	0 0	0 0	0 0	0 0	0 0	0 0	0 0	0 0	0 0	0 0	0 0	0 0	0 1	0 1	0 1	0 0	0 1
chr3:18415619	CDS	C	T	0 0	0 0	0 0	0 0	0 0	0 0	0 0	0 0	0 0	0 0	0 0	0 0	0 0	0 1	0 1	0 1	0 0	0 1
chr3:18415852	CDS	G	C	0 0	0 0	0 0	0 0	0 0	0 0	0 0	0 0	0 0	0 0	0 0	0 0	0 0	0 1	0 1	0 1	0 0	0 1
chr3:18415855	CDS	G	A	0 0	0 0	0 0	0 0	0 0	0 0	0 0	0 0	0 0	0 0	0 0	0 0	0 0	0 1	0 1	0 1	0 0	**1 1**
chr3:18416276	CDS	T	C	0 0	0 0	0 0	0 0	0 0	0 0	0 0	0 0	0 0	0 0	0 0	0 0	0 0	0 1	0 1	0 1	0 0	**1 1**
